# Computational prediction and experimental validation identify functionally conserved lncRNAs from zebrafish to human

**DOI:** 10.1038/s41588-023-01620-7

**Published:** 2024-01-09

**Authors:** Wenze Huang, Tuanlin Xiong, Yuting Zhao, Jian Heng, Ge Han, Pengfei Wang, Zhihua Zhao, Ming Shi, Juan Li, Jiazhen Wang, Yixia Wu, Feng Liu, Jianzhong Jeff Xi, Yangming Wang, Qiangfeng Cliff Zhang

**Affiliations:** 1 MOE Key Laboratory of Bioinformatics, Center for Synthetic and Systems Biology, School of Life Sciences, Tsinghua University, Beijing, China; 2 Beijing Advanced Innovation Center for Structural Biology & Frontier Research Center for Biological Structure, School of Life Sciences, Tsinghua University, Beijing, China; 3 Tsinghua-Peking Center for Life Sciences, Beijing, China; 4 Institute of Molecular Medicine, College of Future Technology, Peking University, Beijing, China; 5 Academy for Advanced Interdisciplinary Studies, Peking University, Beijing, China; 6 State Key Laboratory of Membrane Biology, Institute of Zoology, Chinese Academy of Sciences, Beijing, China; 7 Institute for Stem Cell and Regeneration, Chinese Academy of Sciences, Beijing, China; 8 Department of Biomedical Engineering, College of Future Technology, Peking University, Beijing, China; 9 University of Chinese Academy of Sciences, Beijing, China; 10 School of Life Sciences, Shandong University, Qingdao, China

**Keywords:** Computational biology and bioinformatics, Biological techniques, Functional genomics

## Abstract

Functional studies of long noncoding RNAs (lncRNAs) have been hindered by the lack of methods to assess their evolution. Here we present lncRNA Homology Explorer (lncHOME), a computational pipeline that identifies a unique class of long noncoding RNAs (lncRNAs) with conserved genomic locations and patterns of RNA-binding protein (RBP) binding sites (coPARSE-lncRNAs). Remarkably, several hundred human coPARSE-lncRNAs can be evolutionarily traced to zebrafish. Using CRISPR–Cas12a knockout and rescue assays, we found that knocking out many human coPARSE-lncRNAs led to cell proliferation defects, which were subsequently rescued by predicted zebrafish homologs. Knocking down coPARSE-lncRNAs in zebrafish embryos caused severe developmental delays that were rescued by human homologs. Furthermore, we verified that human, mouse and zebrafish coPARSE-lncRNA homologs tend to bind similar RBPs with their conserved functions relying on specific RBP-binding sites. Overall, our study demonstrates a comprehensive approach for studying the functional conservation of lncRNAs and implicates numerous lncRNAs in regulating vertebrate physiology.

## Abstract (web summary)

A new computational method coupled with a CRISPR–Cas12a screen identifies human long noncoding RNAs (lncRNAs) that lead to cell proliferation defects, which can be rescued by zebrafish homologs. Knockdown of four zebrafish lncRNAs that perturb embryonic development can be rescued by human homologs.

## Main

A major advance in molecular biology and genomics over the last few decades is the discovery and characterization of long noncoding RNAs (lncRNAs), transcripts that are larger than 200 nucleotides (nt) without protein-coding potential^1^. LncRNAs can act as regulators in numerous physiological processes and diseases^2–4^. A well-known example is Xist, which reshapes chromatin architecture to ensure X-chromosome inactivation and achieve dosage compensation in mammalian females^5^. Another example is JPX, which controls the genome-wide binding of CCCTC-binding factor to regulate the 3D structure of the mouse genome^6^. In addition, Bvht has been shown as essential for cardiovascular lineage commitment^7^ and Pnky to regulate the differentiation of neural stem cells^8^.

Dysregulation of lncRNAs has been linked to diverse pathological processes^9,10^. HOTAIR and MALAT1 have been reported to regulate tumorigenesis in various human cancers^11–14^. Mhrt functions in the pathogenesis of cardiomyopathy including hypertrophy and heart failure^15^. A highly conserved lncRNA NORAD functions in maintaining genome stability by sequestering PUMILIO proteins^16^. Despite these notable examples, the function of most lncRNAs remains unknown, and it has been postulated that many lncRNAs may not be functional, owing to their minimal sequence conservation^17^.

Comparative sequence analysis can provide useful information for dissecting lncRNA evolution and functions^18–20^. Through sequence analysis, a study identified THORLNC as a highly conserved lncRNA in vertebrates^21^. Further analysis revealed its conserved oncogenic function in human and zebrafish. Another study reported that defects in zebrafish deficient for the lncRNAs Cyrano and Megamind can be rescued upon complementation with human or mouse homologs^22^. These examples demonstrate the feasibility of searching for functionally conserved lncRNAs through sequence analysis.

However, an overwhelming majority of lncRNAs show little sequence similarity^1,23,24^. For example, only 5.1% of lncRNAs from zebrafish were found to have mammalian homologs in the aforementioned study at the sequence level^22^. Serendipitously, lncRNAs lacking apparent sequence conservation may still have conserved functionality. For example, human JPX can rescue the defects of cell viability in *Jpx* knockout (KO) mouse embryonic stem cells, despite the substantial sequence and structural divergence between the two homologs^25^. It thus appears clear that lncRNA evolution and protein-coding gene evolution have substantially different constraints^2,26,27^. Accordingly, an innovative strategy to identify lncRNA homologs in distant species is urgently needed.

A previous strategy integrating synteny, microhomology of short sequence motifs and secondary structure successfully identified roX homologs among 35 fly species, even for the most distantly related species with no detectable primary sequence similarity^28^. In that study, the microhomology analysis was based on the roX box motif, an essential functional element of roX. In general, lncRNAs often interact with RNA-binding proteins (RBPs) through short sequence motifs to exert their functions^29,30^. Recall, for example, that NORAD functions by binding PUMILIO^16^ and THORLNC functions by binding IGF2BP1 (ref. ^21^). For lncRNA homologs with similar functions, the order and the sequence of these functional elements may appear conserved under selection pressure, whereas other nonessential sequences may evolve rapidly. It should thus be possible to identify functionally conserved lncRNAs across species by evaluating lncRNAs based on overall patterns of conserved RNA motifs.

Here we developed a computational method to identify lncRNAs with conserved genomic locations and patterns of RBP-binding sites across species (coPARSE-lncRNAs). We identified 570 human coPARSE-lncRNAs with a predicted zebrafish homolog, only 17 of which have detectable sequence similarity between the two species. Furthermore, we performed a CRISPR–Cas12a KO screen and identified 75 coPARSE-lncRNAs that promote cell proliferation in at least one of three cancer cell lines. We show that the loss of four human coPARSE-lncRNAs can be phenotypically rescued by their predicted zebrafish homologs and vice versa. We also verified that human, mouse and zebrafish homologs of two coPARSE-lncRNAs interact with similar sets of RBPs, supporting their functional conservation in RBP binding. Importantly, wild-type homologous lncRNA fragments but not variants containing mutated binding sites of certain RBPs rescued the knockdown/KO of a coPARSE-lncRNA in another species, supporting that coPARSE-lncRNAs are functionally related through interactions with specific RBPs. Together, our study substantially expands the known repertoire of conserved lncRNAs across vertebrates, reveals insights about the evolution and mechanisms of lncRNA functions and provides a powerful tool and analytical framework to support further studies of functional lncRNA conservation.

## Results

### LncRNAs across vertebrates share little sequence conservation

**Fig. 1:**
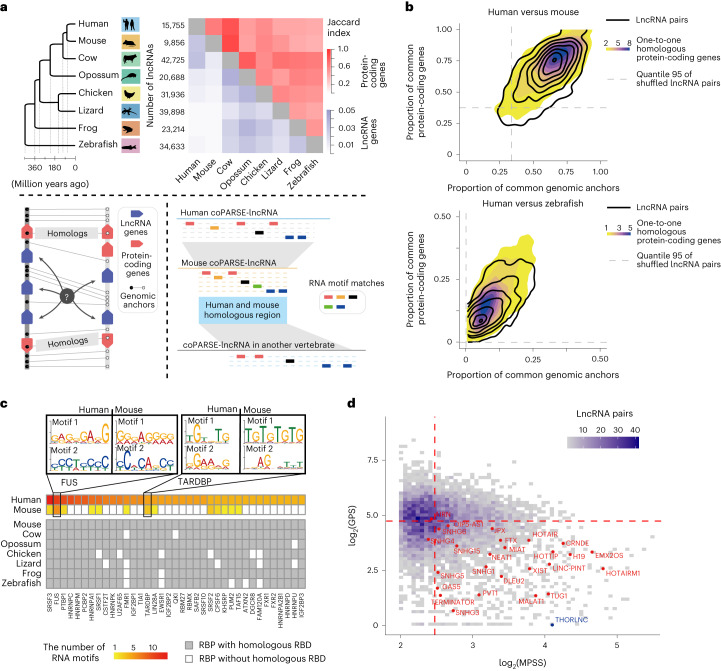
Identification of coPARSE-lncRNA and their homologs across vertebrates. **a**, A simplified workflow for lncHOME analysis of vertebrate lncRNAs. The phylogenetic tree shows the evolutionary descent of eight vertebrates, with the number of annotated lncRNAs in each species. The heatmap shows the Jaccard index of lncRNAs and protein-coding genes identified by sequence similarity across eight vertebrates (top). lncHOME defines coPARSE-lncRNAs by combining the alignment of homologous protein-coding genes and corresponding genomic anchors (bottom left) and analysis of similar motif distribution patterns (bottom right). **b**, Contour line plot of syntenic lncRNAs in human versus mouse and human versus zebrafish identified by lncHOME, in terms of the proportion of common protein-coding genes and the proportion of corresponding genomic anchors. Background density plot showing the proportion scores for protein-coding genes with one-to-one homology. **c**, The distribution of curated RNA motifs for representative RBPs. Represented motifs for two example RBPs (FUS and TARDBP) are shown. **d**, coPARSE-lncRNA homolog pairs with similar motif distribution patterns between human and mouse. A coPARSE-lncRNA with annotation in the lncRNAdb database is highlighted in red. The lncRNA THORLNC is highlighted in blue. Red dashed lines represent the median value of the MPSSs and the GPSs.

**Extended Data Fig. 1:**
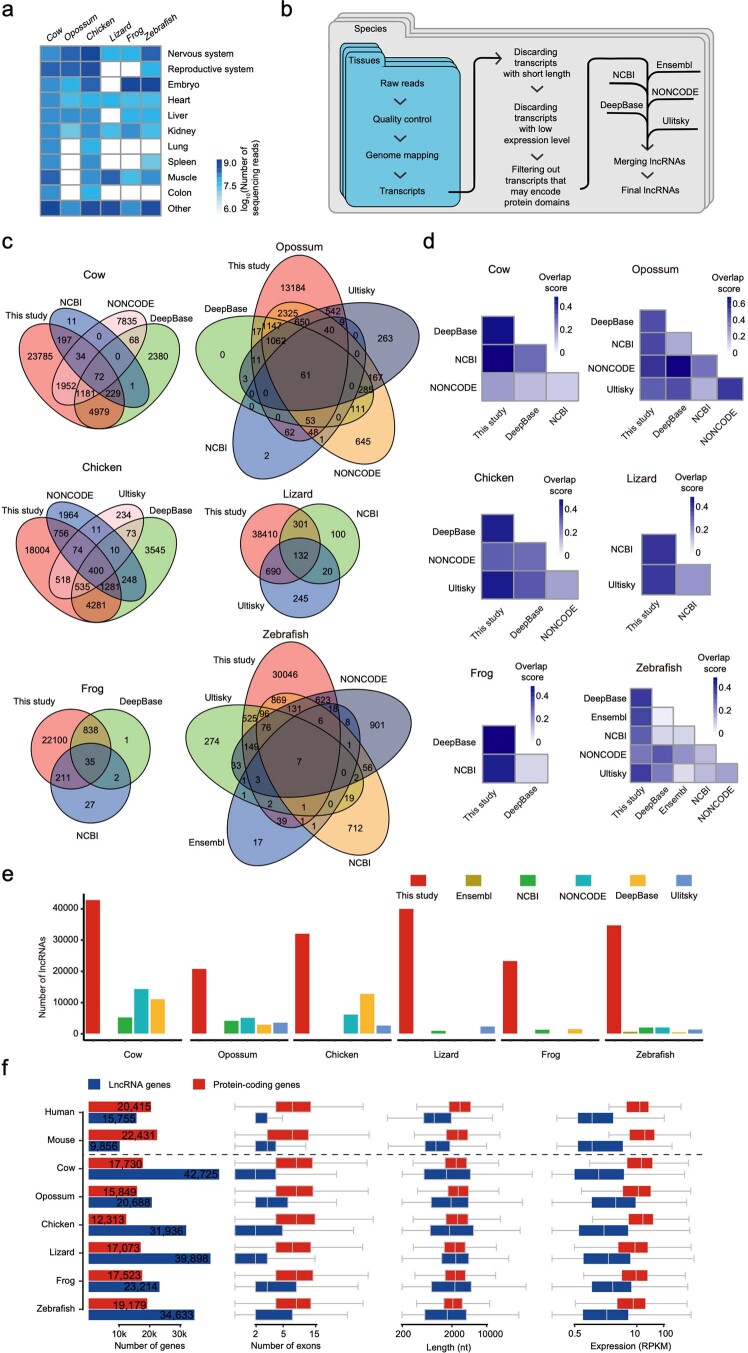
Curation of lncRNAs. **a**, RNA-seq data collected for lncRNA annotation. In total, more than 12 billion sequencing reads were collected for six vertebrates. **b**, Pipeline for lncRNA curation. **c**, Venn diagram showing the overlap of curated lncRNAs of the six species in this study with annotated lncRNAs from the indicated sources. **d**, Heatmaps showing the overlap scores among curated lncRNAs of the six species in this study and annotated lncRNAs from other indicated sources. **e**, Comparison of the number of lncRNA genes in our study vs. previously reported lncRNA datasets. **f**, The number of genes and the distribution of number of exons, lengths, and expression levels of protein-coding genes and lncRNAs curated in this study. Boxes, IQR. Centre lines, median. Whiskers, values within 1.5 × IQR of the top and bottom quartiles.

To explore lncRNA homology, we initially annotated lncRNA datasets for six vertebrates, including cow, opossum, chicken, lizard, frog and zebrafish, as an addition to the existing high-quality lncRNA annotations for human and mouse from the GENCODE project^31^ (Fig. 1a; Methods). Specifically, we collected 233 RNA-sequence (RNA-seq) datasets for these six vertebrates (Extended Data Fig. 1a and Supplementary Table 1). We then assembled transcripts from the RNA-seq data and identified lncRNAs adapting an established pipeline^24^, where we filtered out transcripts with protein-coding potential >0.5 predicted by the coding-potential assessment tool (CPAT)^32^ (Extended Data Fig. 1b). We found that our curated lncRNAs share extensive overlap with the lncRNAs from five other public sources, including Ensembl^33^ and a curation from the Ulitsky laboratory^24^ (Extended Data Fig. 1c,d). We then merged our annotations with these public curations to form the final lncRNA dataset (Extended Data Fig. 1e,f).

**Extended Data Fig. 2:**
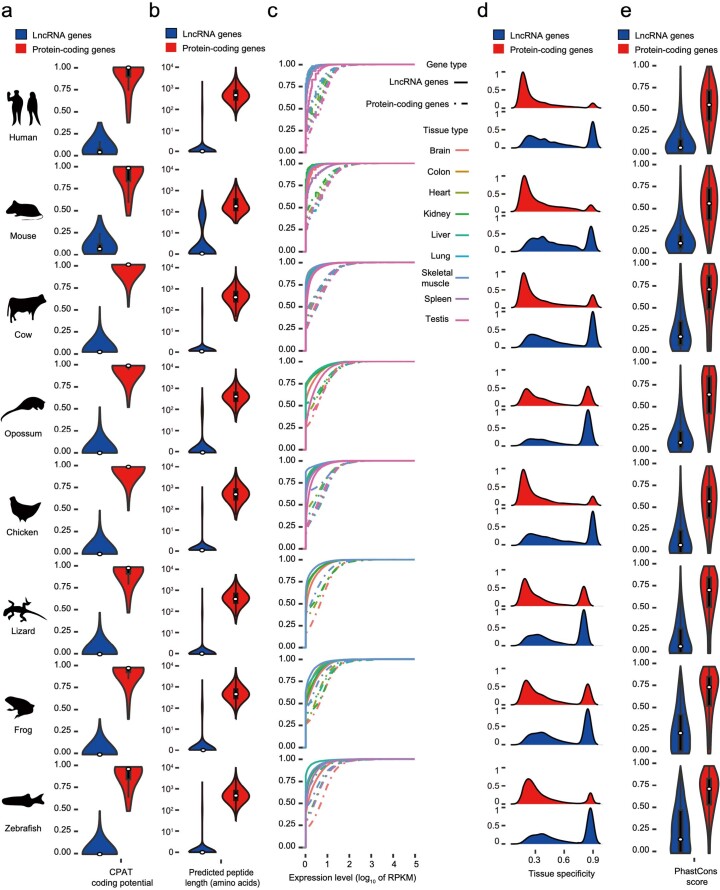
Assessment of lncRNA annotations. **a**, Protein-coding potential (estimated by CPAT) of the curated lncRNAs compared to protein-coding genes. **b**, Predicted peptide lengths of the curated lncRNAs compared to protein-coding genes. **c**, Cumulative distribution of the expression level of the curated lncRNAs compared to protein-coding genes. **d**, Distribution of tissue expression specificity scores of the curated lncRNAs compared to protein-coding genes. **e**, PhastCons conservation scores of the curated lncRNAs compared to protein-coding genes. The numbers of curated lncRNAs and protein-coding genes are consistent with Extended Data Fig. 1f. For panels a, b, and e, boxes, IQR. Centre lines, median. Whiskers, values within 1.5 × IQR of the top and bottom quartiles.

We obtained 20,688–42,725 candidate lncRNAs for the six vertebrate species (Fig. 1a and Extended Data Fig. 1e,f). Agreeing with previous reports^20,24^, these lncRNAs showed consistently lower protein-coding potential, lower expression level and higher tissue specificity than protein-coding genes (Extended Data Fig. 2a–d). As expected, there was very little sequence conservation among the lncRNAs across these vertebrates (Fig. 1a and Extended Data Fig. 2e). From a pairwise BLAST analysis between the eight vertebrates, only 0.3–3.9% of the lncRNAs from one species had detectable sequence similarity with lncRNAs from another species (Methods), levels much lower than those for protein-coding genes (40–90%). Collectively, these results reinforce the concept that lncRNAs generally share very low sequence-level conservation.

### Identification of candidate lncRNA homologs with synteny

**Extended Data Fig. 3:**
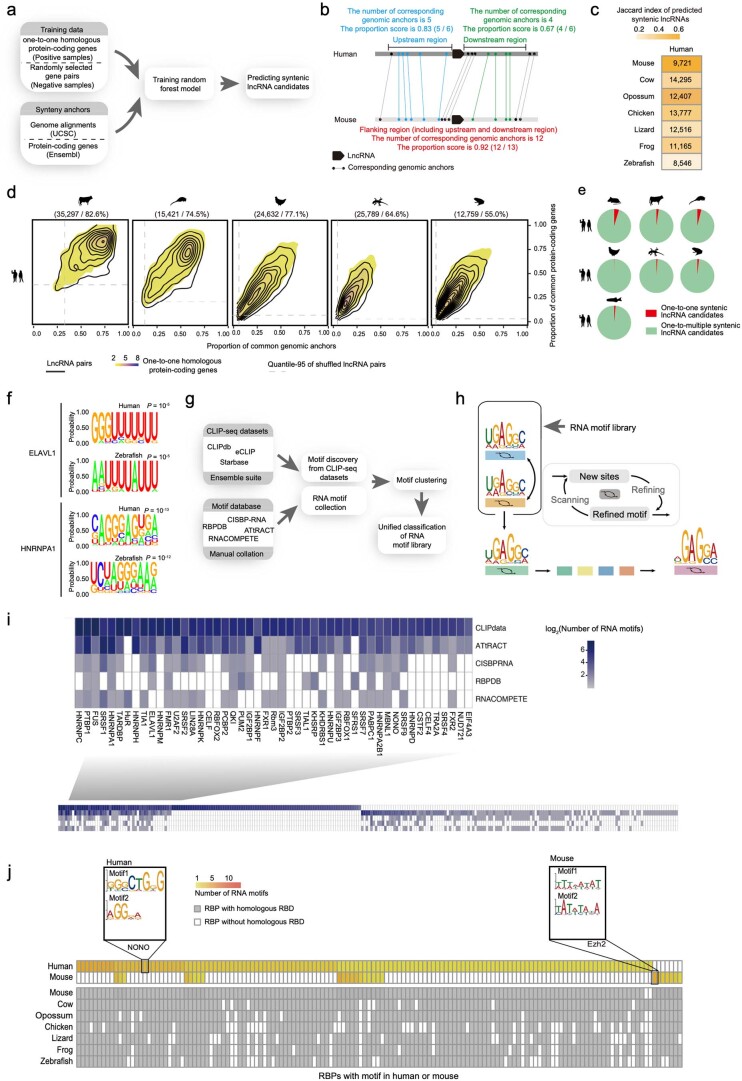
Identification of syntenic lncRNAs across species and curation of RNA motifs from CLIP-seq datasets and public motif databases. **a**, Pipeline for syntenic lncRNA identification. A random forest model was trained to predict syntenic lncRNAs between each pair of species based on the two defined sets of ‘synteny indicators’, using one-to-one homologs of protein-coding genes as positive samples and randomly selected gene pairs as negative samples for model training. **b**, The calculation of 6 features (the numbers and the proportion scores) for the corresponding genomic anchors in the upstream region, the downstream region, or the flanking region of one pair of human and mouse lncRNAs. **c**, Heatmap showing the numbers and Jaccard index values for predicted syntenic lncRNAs between human and the seven indicated species. **d**, Contour line plot of syntenic lncRNAs for human vs. five other species identified by lncHOME, in terms of the proportion of common protein-coding genes and the proportion of corresponding genomic anchors. The background density plot shows the same proportion scores for protein-coding genes with one-to-one homology. **e**, Pie plots showing the proportions of human lncRNAs with one-to-one syntenic lncRNAs (red) and one-to-multiple syntenic lncRNAs in another species (green). **f**, The sequence motifs of ELAVL1 and HNRNPA1 in human and zebrafish called from the binding sites from the CLIP data. *P* values were calculated by one-sided Binomial test. **g**, Pipeline for RNA motif curation for human and mouse. RNA motifs were identified in the CLIP-seq datasets using the MEME suite, and collected from public databases (*that is*, RNACOMPETE, CIS-RBP, RBPDB, and ATtRACT). Motif clustering was performed to merge similar motifs. **h**, Pipeline for RNA motif extrapolation across species. The RNA motifs curated for human and mouse were used to scan for motif matches in the transcriptomes of another species. Then the motif matches were used to update (or refine) the original motif to generate new motifs for the other species. **i**, The number of curated human RNA motifs. **j**, The distribution of curated RNA motifs for representative RBPs. Represented motifs for two example RBPs (NONO and Ezh2) are shown.

Synteny analysis can identify chunks of genomic regions sharing the same evolutionary origin^18^. We speculated that synteny information may be informative for identifying conserved lncRNAs. Pursuing this, we designed a predictive random forest model to identify candidate lncRNA homologs across vertebrates for each human lncRNA based on synteny (Fig. 1a and Extended Data Fig. 3a; Methods). We used two sets of ‘synteny indicators’ along the genomes and defined 12 features of these two ‘synteny indicators’ for random forest model prediction (Extended Data Fig. 3b). The protein-coding homolog pairs and their associated scores were used as the training set for the model, which was then used for predicting synteny relationship of lncRNA pairs.

This analysis discovered syntenic counterparts in other species for thousands of human lncRNA genes (Extended Data Fig. 3c and Supplementary Table 2). The genome context for the identified syntenic lncRNA candidates was largely similar to that of homologous protein-coding genes (Fig. 1b and Extended Data Fig. 3d). Nevertheless, fewer than 10% of lncRNAs had unique syntenic lncRNAs in the seven other species, while most human lncRNAs had 2–5 syntenic candidates (Extended Data Fig. 3e). Thus, further analysis is needed to refine the list of candidates to identify evolutionarily conserved lncRNA homologs.

### Identification of evolutionarily coPARSE-lncRNA homologs

RBPs function as essential regulators of RNA, and recent studies have accumulated large-scale data resources for transcriptome-wide profiling of RBP-binding sites^34–36^. Numerous studies have observed that RBP–RNA interactions tend to be conserved across species^37,38^. For instance, binding motifs of ELAVL1 and HNRNPA1 are similar in human and zebrafish (Extended Data Fig. 3f; Methods). We thus speculated that consensus patterns of RBP-binding sites could be informative for identifying functionally conserved lncRNA homologs.

We first defined a library of RBP-binding motifs for the eight species examined in our study (Methods). For humans, we constructed the library based on the following: (1) results of motif calling from high-throughput cross-linking and immunoprecipitation (CLIP)-seq data using the MEME suite^39^ and (2) available RNA motifs from databases including RNACOMPETE^38^, CISBP-RNA^38^, RBPDB^40^ and ATtRACT^41^ (Extended Data Fig. 3g). For each of the other species, we extrapolated every human motif to define a corresponding new species-specific motif, using an iterative mapping-and-refinement strategy (Extended Data Fig. 3h). Finally, we obtained 2,171 motifs for human (181 RBPs), 2,165 motifs for mouse (179 RBPs) and 1,844 motifs for zebrafish (144 RBPs; Fig. 1c, Extended Data Fig. 3i,j and Supplementary Table 3).

**Extended Data Fig. 4:**
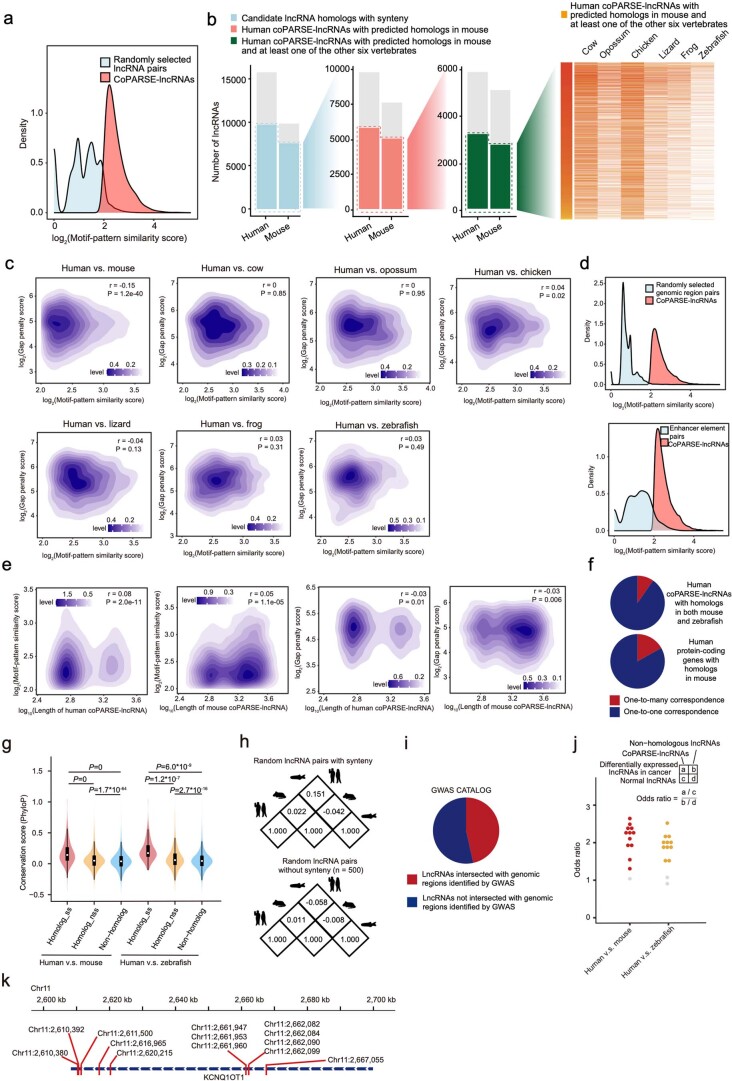
Identification of candidate human coPARSE-lncRNAs with predicted homologs across vertebrates and disease relevance of human coPARSE-lncRNAs. **a**, Density plot of MPSS for randomly selected lncRNA pairs and coPARSE-lncRNAs. **b**, Bar plots showing the number of candidate lncRNA homologs with synteny, human coPARSE-lncRNAs with predicted homologs in mouse, and human coPARSE-lncRNAs with predicted homologs in mouse and at least one of the other six vertebrates (left), and heatmap showing occurrence of the homologs in six other vertebrates (right). **c**, Gradient plots of MPSS and GPS for coPARSE-lncRNA homolog pairs between human and the seven other species. **d**, Density plots of MPSS for randomly selected genomic region pairs (left) and enhancer element pairs (right), compared to coPARSE-lncRNAs. **e**, Gradient plots showing the correlation between lncRNA lengths and MPSS (and GPS) for human and mouse coPARSE-lncRNAs. For panels c and e, r, Pearson correlation coefficient, two-sided Student’s *t*-test. **f**, The percentage of genes with one-to-many correspondence homologs in mouse and in both mouse and zebrafish for human coPARSE-lncRNA genes and protein-coding genes. **g**, Distribution of average conservation scores (PhyloP, n = 605/17 for human v.s. mouse/zebrafish) for coPARSE-lncRNA homolog pairs with sequence similarity (homolog_ss) and without sequence similarity (homolog_nss, n = 4,959/553 for human v.s. mouse/zebrafish), and paired lncRNAs randomly selected from human and mouse lncRNAs (non-homolog, n = 5,000). Two-sided Mann-Whitney U test. Boxes, IQR. Centre lines, median. Whiskers, values within 1.5 × IQR of the top and bottom quartiles. **h**, Correlation of tissue-specific expression score of random lncRNA pairs with genomic synteny or without genomic synteny among three species. Numbers showing mean values from a random sampling (500 times). **i**, Overlaps of coPARSE-lncRNAs with disease-linked genomic regions from the GWAS CATALOG database. **j**, Enrichment of the human coPARSE-lncRNAs with predicted homologs in mouse or zebrafish for differentially expressed human lncRNAs across different cancer types. Each dot represents a cancer type; the orange and yellow colors indicate significant enrichment. *P* values were calculated by two-sided Fisher’s exact test. **k**, Genomic locations of the ClinVar mutations within *KCNQ1OT1*.

We then identified homologous lncRNAs for every human lncRNA based on a motif-pattern similarity score (MPSS) and a gap penalty score (GPS; Methods). We defined ‘lncRNA Homology Explorer (lncHOME)-predicted lncRNA homologs’ as the two members of a lncRNA pair between two vertebrates for which (1) the MPSS was higher than the corresponding background threshold (*P* < 0.05, permutation test; Extended Data Fig. 4a), (2) the GPS was lower than the corresponding background threshold (*P* < 0.05, permutation test) and (3) the MPSS was higher than 0.8 times of the maximum MPSS among all candidate pairs.

The lncHOME pipeline predicted homologs for 570–5,564 human lncRNAs in other vertebrates (Supplementary Tables 4 and 5), which we defined as coPARSE-lncRNAs for their conserved patterns in synteny and RBP-binding sites. Specifically, 5,564 (35.3%) human lncRNAs are coPARSE-lncRNAs with predicted homologs in mouse, among which around a half had predicted homologs in at least a third species, and notably, 570 (3.6%) human coPARSE-lncRNAs had predicted homologs in zebrafish (Extended Data Fig. 4b). We found no correlation between MPSS and GPS (Extended Data Fig. 4c), indicating no inflation of our estimation of significance for the identified coPARSE-lncRNA homolog pairs.

Supporting the accuracy of the pipeline, lncHOME identified the correct mouse homologs of all 26 human lncRNAs in lncRNAdb^9^ with known homologs (Fig. 1d). Additionally, we found that many well-known lncRNAs are coPARSE-lncRNAs. For example, we found that THORLNC^21^ is a coPARSE-lncRNA with a predicted mouse homolog Gm29359.

We examined length-matched, nontranscribed DNA regions or enhancer element pairs that are in the same syntenic regions of coPARSE-lncRNA pairs (Methods) and found that few selected genomic region pairs (0.2%) or enhancer element pairs (1.9%) were predicted as ‘coPARSE’ regions, supporting that lncHOME predictions have a low false positive rate (Extended Data Fig. 4d). We also found no correlations between the lengths of the coPARSE-lncRNAs and MPSS or GPS (Extended Data Fig. 4e).

It bears mention that 515 (90.4%) of 570 human coPARSE-lncRNAs have one-to-one homolog correspondence in both mouse and zebrafish. For comparison, 83.2% of all human protein-coding genes have one-to-one homolog correspondence in mouse (Extended Data Fig. 4f). Together, these results demonstrate that incorporating conserved RBP-binding site data substantially improves the accuracy of lncHOME in predicting potential lncRNA homologs.

### Evolutionary and functional features among lncRNA homologs

**Fig. 2:**
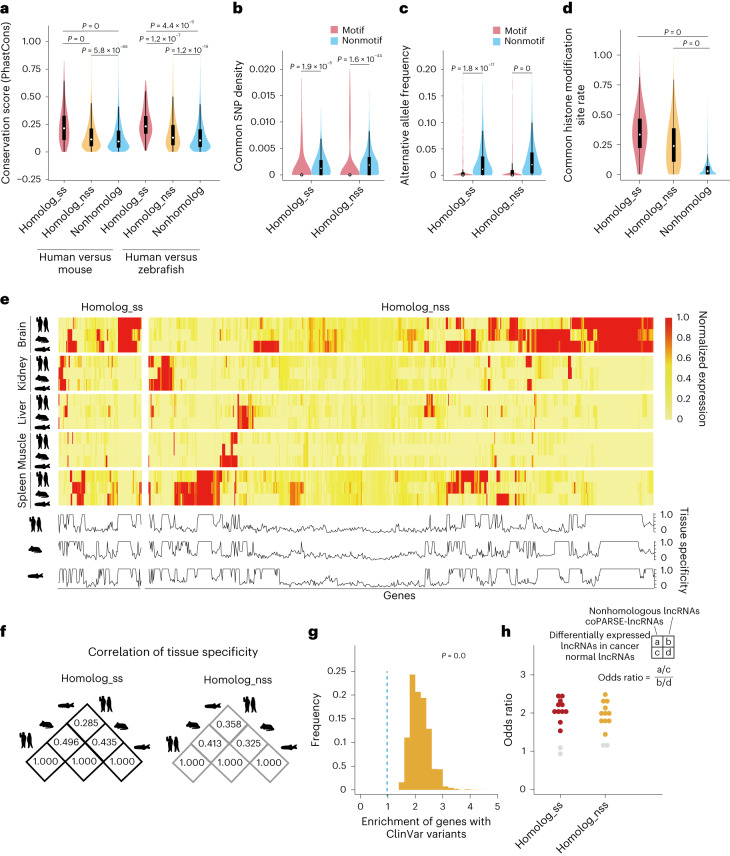
The coPARSE-lncRNAs and their predicted homologs share similar evolutionary and functional features. **a**, The distribution of average conservation scores (PhastCons) for coPARSE-lncRNA homolog pairs with sequence similarity (homolog_ss, *n* = 605/17 for human versus mouse/human versus zebrafish) and without sequence similarity (homolog_nss, *n* = 4,959/553 for human versus mouse/human versus zebrafish), and paired lncRNAs randomly selected from human and mouse lncRNAs (nonhomolog, *n* = 5,000). **b**, The distribution of common SNP density of SNPs in motif or nonmotif regions in human coPARSE-lncRNAs among the homolog_ss (*n* = 605) and homolog_nss (*n* = 4,959) groups of lncRNA pairs. **c**, The distribution of major alternative allele frequency of SNPs in motif or nonmotif regions in human coPARSE-lncRNAs among the homolog_ss (*n* = 605) and homolog_nss (*n* = 4,959) groups of lncRNA pairs. **d**, The distribution of the common histone modification site rate among the homolog_ss (*n* = 605), homolog_nss (*n* = 4,959) and nonhomolog (*n* = 5,000) groups of lncRNA pairs. For **a**–**d**, two-sided Mann–Whitney *U* test. Boxes, IQR. Center lines, median. Whiskers, values within 1.5× IQR of the top and bottom quartiles. **e**, Heatmap of normalized expression values of coPARSE-lncRNAs and their predicted homologs in five organs (brain, kidney, liver, muscle and spleen) and three species (human, mouse and zebrafish) are displayed (top), and distribution of tissue-specific expression score (among the five organs) of the coPARSE-lncRNAs and their homologs (bottom). **f**, Correlation of tissue specificity of homolog_ss and homolog_nss groups of coPARSE-lncRNAs and their homologs among three species. **g**, Distribution of enrichment for human coPARSE-lncRNA genes with ClinVar mutations (excluding the mutations falling in exons of protein-coding genes), compared to randomly selected lncRNA genes (*P* value calculated using a permutation test). Blue dashed lines represent the nonenrichment threshold of 1. **h**, Enrichment of the homolog_ss and homolog_nss groups of human coPARSE-lncRNAs with homologs in mouse for differentially expressed lncRNAs across different cancer types. Each dot represents a cancer type, and the orange and yellow colors indicate significant enrichment (*P* values calculated using two-sided Fisher’s exact test). IQR, interquartile range.

We divided the coPARSE-lncRNAs and their homologs into the following two groups: a homolog_ss group containing 605 coPARSE-lncRNA homolog pairs with high sequence similarity (>50%) and a homolog_nss group containing the other 4,959 coPARSE-lncRNA homolog pairs with low or no sequence similarity. We then compared sequence conservation for the coPARSE-lncRNA homolog pairs in the two groups (Methods). For both human versus mouse and human versus zebrafish, the homolog_ss coPARSE-lncRNAs are substantially more conserved than the homolog_nss coPARSE-lncRNAs, whereas the homolog_nss coPARSE-lncRNAs were only marginally more conserved than random lncRNAs, based on the PhastCons and PhyloP conservation scores^42,43^ (Fig. 2a and Extended Data Fig. 4g). Interestingly, we found that the motif regions have a much lower density of common single-nucleotide polymorphisms (SNPs) or major alternative allele frequencies (Fig. 2b,c) than nonmotif regions for both homolog_ss and homolog_nss coPARSE-lncRNAs. These results suggest that predicted motif regions of coPARSE-lncRNAs have undergone stronger selection pressures than the nonmotif regions.

We also found that coPARSE-lncRNA homologs share a relatively higher level of histone modification pattern similarity than the random lncRNA pairs (Fig. 2d; Methods), suggesting similar transcription programs regulating coPARSE-lncRNA homolog pairs. Indeed, coPARSE-lncRNA homolog pairs exhibit comparable tissue-expression profiles across different species, both higher than other random syntenic lncRNA pairs (Fig. 2e,f and Extended Data Fig. 4h).

Moreover, 270 (47%) of 570 human coPARSE-lncRNAs located in genomic regions implicated in diseases by genome-wide association studies, a proportion higher than that of other human lncRNAs (Extended Data Fig. 4i). It is also notable that compared to random lncRNAs, the human coPARSE-lncRNAs are enriched for disease-associated mutations (Fig. 2g), and their expression is more likely to be dysregulated in cancer tissues (Fig. 2h and Extended Data Fig. 4j). As an illustration, we noted 13 ClinVar^44^ mutations within KCNQ1OT1 (Extended Data Fig. 4k), a coPARSE-lncRNA that has been previously linked to Beckwith–Wiedemann syndrome^45^.

### A CRISPR screen identified lncRNAs promoting proliferation

**Fig. 3:**
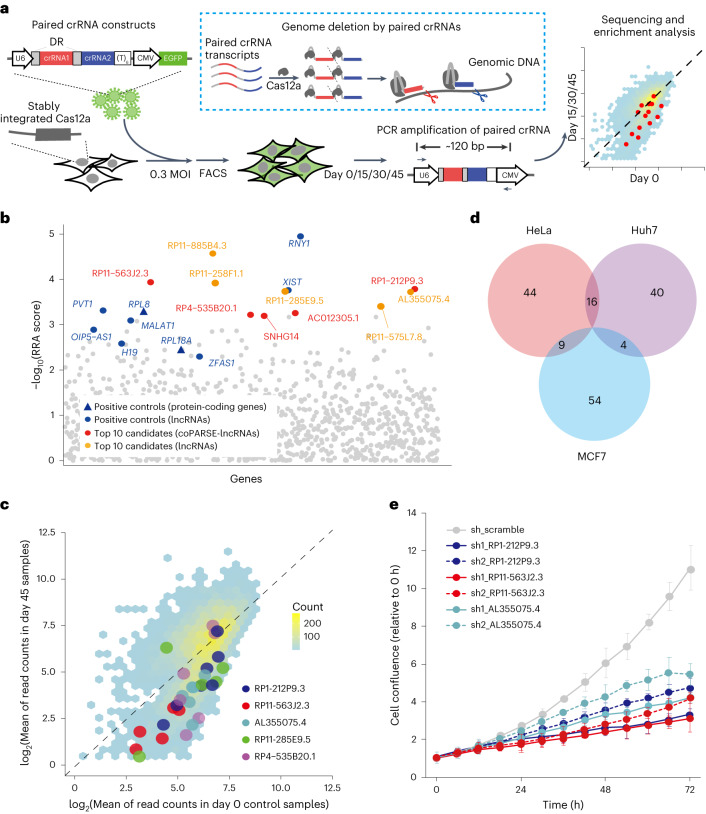
CRISPR–Cas12a screening and validation of coPARSE-lncRNA functions. **a**, The crRNA library was delivered into cells stably expressing Cas12a by lentiviral infection. Infected cells were collected by fluorescence-activated cell sorting (FACS; green fluorescence). For screening, cells were cultured for 15–45 d before genome DNA extraction and high-throughput sequencing analysis of the barcoded crRNA regions. Each DNA oligonucleotide sequence encodes two crRNAs (represented in red and blue), which will be transcribed and processed to generate individual mature crRNAs by Cas12a; these mature crRNAs will guide Cas12a to cut target genome regions. DR (19 nt). **b**, The RRA scores for the top-ranking negatively selected lncRNAs. Note that smaller RRA scores indicate a stronger selection of the corresponding lncRNAs. The coPARSE-lncRNAs of the top ten negatively selected lncRNAs are highlighted in red, whereas the non-coPARSE-lncRNAs are highlighted in orange. Nine positive control genes are shown in blue (round dots for lncRNAs and triangles for protein-coding genes). Background represents the overall distribution. **c**, The mean read count value for paired crRNAs at day 45 relative to that of day 0 for lncRNA genes in our screening library. Highlighted dots are paired crRNAs for five negatively selected candidate genes in our screening assay, and the background represents the overall distribution. **d**, Overlap of the negatively selected lncRNAs in the three indicated cell lines. **e**, Cell proliferation validation assays in HeLa cells treated with two independent shRNAs for each candidate lncRNA. Error bars, means ± s.d., *n* = 3 biologically independent experiments. DR, direct repeats.

**Extended Data Fig. 5:**
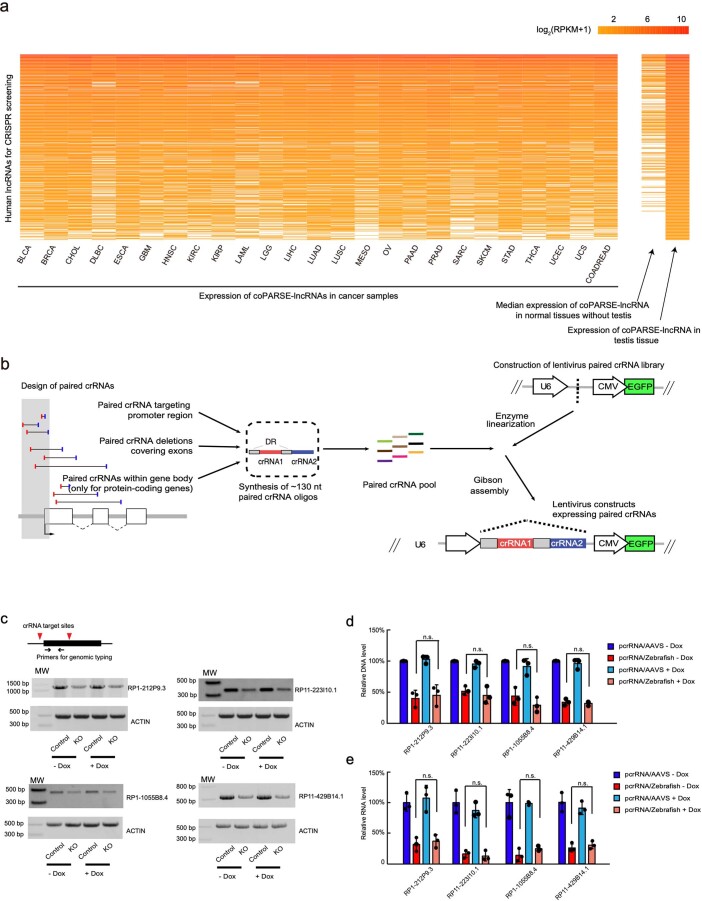
Selection of lncRNA candidates for CRISPR-Cas12a knockout screening. **a**, Heatmaps showing the expression of the lncRNA candidates across various cancer samples (left), and the median expression of the indicated lncRNAs in normal tissues excluding testis and in testis tissues (right). TCGA cancer types include BLCA, BRCA, CHOL, DLBC, ESCA, GBM, HNSC, KIRC, KIRP, LAML, LGG, LIHC, LUAD, LUSC, MESO, OV, PAAD, PRAD, SARC, SKCM, STAD, THCA, UCEC, UCS, COADREAD (left to right). **b**, Pipeline for crRNA library design and construction. **c**, lncRNA gene knockout strategy and location of primers used for genome PCR. Representative images of genomic DNA PCR amplification of the four indicated lncRNA genes. MW: DNA marker. n = 3 independent biological experiments. **d**, Quantitative analysis of RP1-212P9.3, RP11-223I10.1, RP1-1055B8.4 and RP11-429B14.1 knockout efficiency based on genome PCR results. The genomic DNA amplified by PCR was first normalized to *ACTB* and then to HeLa cells treated with control AAVS without addition of Dox. Error bars, means ± SD, n = 3 biologically independent experiments, two-tailed Student’s *t* test, n.s., not significant. **e**, RT-qPCR analysis of the lncRNA expression level of RP1-212P9.3, RP11-223I10.1, RP1-1055B8.4 and RP11-429B14.1 in the knockout and complemented HeLa cells. Error bars, means ± SD, n = 3 biologically independent experiments, two-sided Student’s *t* test, n.s., not significant. Source data

To functionally characterize coPARSE-lncRNAs, we performed an extensive CRISPR-based KO screen (Methods). Briefly, we conducted cell proliferation assays using cancer cell lines for 574 human lncRNAs (including 249 coPARSE-lncRNAs with predicted homologs in zebrafish) that are highly expressed in human cancer samples (Extended Data Fig. 5a and Supplementary Table 6). We used the nuclease Cas12a^46^, coupled with a pair of crRNA oligonucleotide sequences, to generate genome deletions to KO the function of target genes (Fig. 3a). To construct the KO library, we designed 20 pairs of crRNA oligonucleotide sequences for each of the 574 lncRNAs to purposely target regions including promoter regions^47^. We then constructed a library based on a lentiviral vector containing paired crRNAs driven by the U6 promoter with a downstream reporter cassette of cytomegalovirus promoter-enhanced green fluorescent protein (CMV–EGFP) ^47^ (Extended Data Fig. 5b).

**Extended Data Fig. 6:**
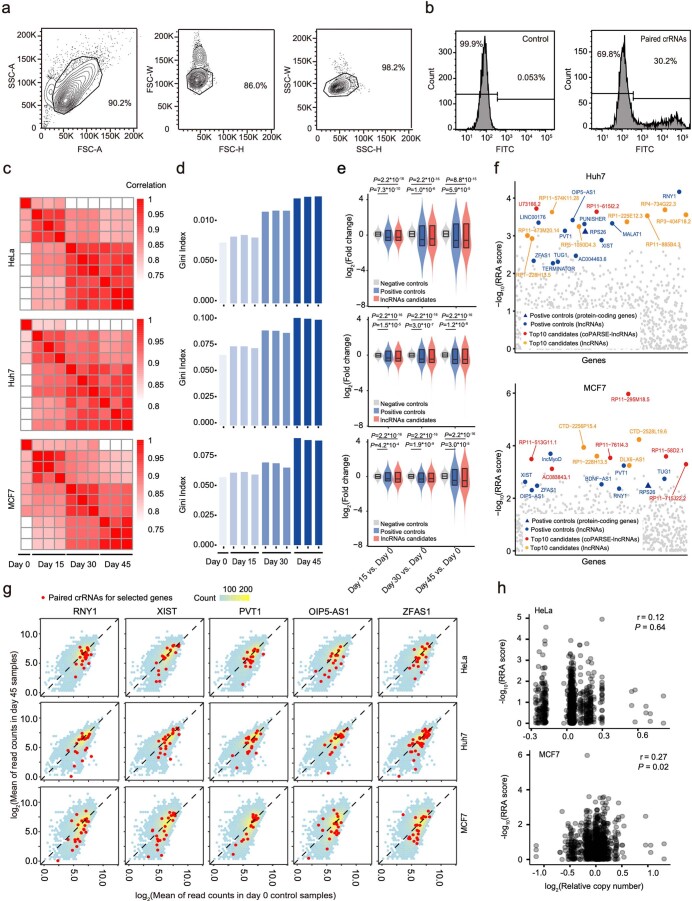
CRISPR-Cas12a screening and validation of coPARSE-lncRNAs with cell proliferation function. **a**, Representative contour plots of FITC FACS gating strategy. Cells were separated from debris based on the forward scatter area and side scatter area. Two polygon gates were applied using the width and height metrics of the side scatter and forward scatter. FITC signals are shown for all live singlets. **b**, Control HeLa cells stably expressed Cas12a have no green fluorescence signal. The populations with FITC positive signal are the knockout cells with paired crRNAs targeting coPARSE-lncRNAs. **c**, **d**, Correlation (**c**) and Gini index (**d**) of the screening sample replicates of the three indicated cell lines. **e**, Distribution of the fold changes of the paired crRNAs targeting negative controls (n = 100 for three cells), positive controls (n = 1,700 for HeLa and Huh7 cells, n = 1,697 for MCF7 cell), and candidate lncRNAs (n = 9,594 for HeLa cell, n = 9,596 for Huh7 cell, n = 9,587 for MCF7 cell). Two-sided Student’s *t*-test. Boxes, IQR. Centre lines, median. **f**, RRA scores of the top-ranking negatively selected lncRNAs calculated for Huh7 and MCF7 cells. Positive control genes that are negatively selected are shown in blue (round dots for lncRNAs and triangles for protein-coding genes). The coPARSE-lncRNAs of the top ten negatively selected lncRNAs are highlighted as red dots, as non-coPARSE-lncRNAs are highlighted as orange dots. **g**, The mean read count value for paired crRNAs at day 0 and day 45. Highlighted dots are paired crRNAs for the five negatively selected candidate genes; the background of gray to yellow density represents overall distribution. **h**, Correlation of the RRA scores of the lncRNAs in our screening and relative copy number data from ENCODE for HeLa and MCF7 cells. R, Pearson correlation coefficient, two-sided Student’s *t*-test.

The PCR results indicated that the KO efficiency ranged from 47.2% to 71.0% for the targeted regions (Extended Data Fig. 5c,d). The real-time quantitative PCR (RT–qPCR) analysis indicated 57.9–87.5% KO efficiency for the examined lncRNAs (Extended Data Fig. 5e). We introduced the library by lentiviral transduction to three cancer cell lines (HeLa, Huh7 and MCF7) stably expressing Cas12a and selected green fluorescent protein (GFP)-positive cells for propagation (Extended Data Fig. 6a,b). We observed high agreement between experimental replicates (Extended Data Fig. 6c) and high evenness of the crRNA distribution at day 0 as well as a gradual increase in unevenness during screening (Extended Data Fig. 6d). As expected, the overall abundance of crRNAs targeting positive controls consistently decreased during screening, as compared with the crRNAs targeting the nonfunctional adeno-associated virus integration site 1 (AAVS1) intron loci (Extended Data Fig. 6e). Collectively, these data provide compelling evidence for the robustness and reliability of our KO screen.

We identified 167 lncRNAs (75 coPARSE-lncRNAs) with significantly decreased crRNA abundance at days 15, 30 and 45 as compared to day 0 in the three cancer cell lines (Fig. 3b–d, Extended Data Fig. 6f,g and Supplementary Table 7). The screen recovered 74% or 14 positive control oncogenes (for example, *XIST*^48^ and *RNY1*; Fig. 3b and Extended Data Fig. 6g). Notably, 82% of the crRNAs targeting these genes were depleted (Supplementary Table 7). Consistent with a previous study^47^, we observed limited overlap between different cell lines (Fig. 3d). Notably, there is no correlation between robust rank aggregation (RRA) scores and genomic copy-number variation (CNV), indicating that the screening results were not biased by copy-number-amplified regions (Extended Data Fig. 6h), which is a potential cause for false positives in CRISPR screening^49^.

**Extended Data Fig. 7:**
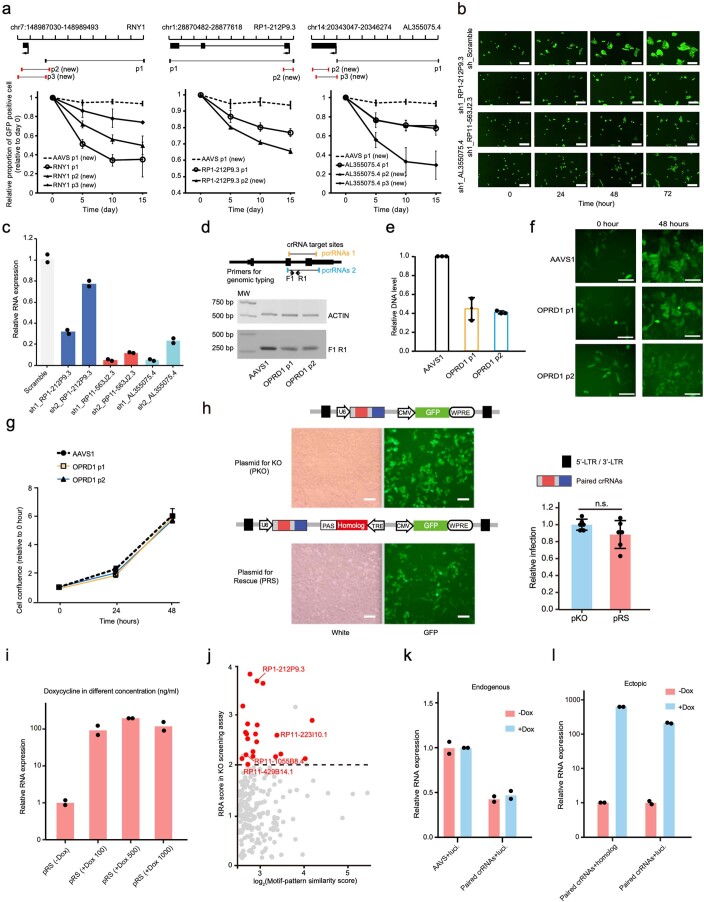
Confirmation of cell proliferation-promoting function for coPARSE-lncRNAs identified by CRISPR-Cas12a screening and the selection of coPARSE-lncRNA candidates for knockout-rescue assay. **a**, Effects of crRNAs targeting the positive control gene *RNY1* (left), RP1-212P9.3 (middle) and AL355075.4 (right) on cell proliferation in HeLa cells. Relative confluence of cell proliferation was calculated by normalizing GFP positive percentages at the indicated time points relative to control (day 0). Newly designed paired crRNAs not in the original library are marked with ‘new’. **b**, Culture images of cell proliferation validation assays for HeLa cells treated with two independent shRNAs for each candidate coPARSE-lncRNA. Scale bars, 200 μm. The experiments were repeated three times with similar results. **c**, Relative RNA expression of RP1-212P9.3, RP11-563J2.3, and AL355075.4 in different shRNA knockdown assays. **d**, Design of the crRNA pairs and the PCR primers. Representative images of genomic DNA PCR amplification are shown. *ACTIN* was used as reference. MW: DNA marker. n = 3 independent biological experiments. **e**, Knockout efficiency based on PCR results for the targeted genome regions of OPRD1. The genomic DNA amplified by PCR was first normalized to the *ACTB* locus and then to HeLa cells treated with control *AAVS1* crRNAs. **f**, Representative cell proliferation images for GFP-positive cells. Note that the selected images are all from a fixed field of view. Scale bars, 100 μm. The experiments were repeated three times with similar results. **g**, High-content imaging assay. The relative cell confluence at the indicated time points was calculated by normalizing to the cell numbers of day 0. **h**, Comparison of lentivirus packaging efficiency for the original knockout and the knockout-rescue plasmids. The lentivirus packaging efficiency was measured as the infection rates at 3 days post lentivirus infection. Scale bars, 200 μm, Error bars, means ± SD, n = 6 biologically independent experiments, two-sided Student’s *t*-test, n.s., not significant. **i**, Successful induction of ectopic gene expression under the indicated induction conditions. **j**, Scatter plot of the candidate coPARSE-lncRNAs (highlighted in red) selected for the knockout-rescue assay. **k**, **l**, Relative RNA expression of targeting genes for the knockout-rescue assays. Two pairs of primers were used to detect (**k**) endogenous and (**l**) ectopic expression of RP1-212P9.3 and its homolog or luciferase fragment. For panels a, e, and g, error bars, means ± SD, n = 3 independent biological experiments. For panels c, i, k, and l, n = 2 independent biological experiments. Source data

We focused on several negatively selected coPARSE-lncRNAs to validate the screening results. We confirmed that, for a positive control lncRNA RNY1 and two candidate coPARSE-lncRNAs (RP1-212P9.3 and AL355075.4), KO by all paired crRNAs caused a substantial reduction in the cell proliferation rate (Extended Data Fig. 7a). Of particular note, shRNA knockdown of three coPARSE-lncRNAs (RP1-212P9.3, AL355075.4 and RP11-563J2.3) confirmed their functions in promoting cell proliferation (Fig. 3e and Extended Data Fig. 7b,c). Additionally, KO of the protein-coding gene *OPRD1*, which partially overlaps with RP1-212P9.3, did not affect cell proliferation (Extended Data Fig. 7d–g), supporting that the lncRNA gene per se, but not its adjacent protein-coding gene *OPRD1*, promotes cell proliferation. Thus, our screen has identified conserved coPARSE-lncRNAs regulating cancer cell proliferation.

### Functional validation of the conservation of lncRNA homologs

**Fig. 4:**
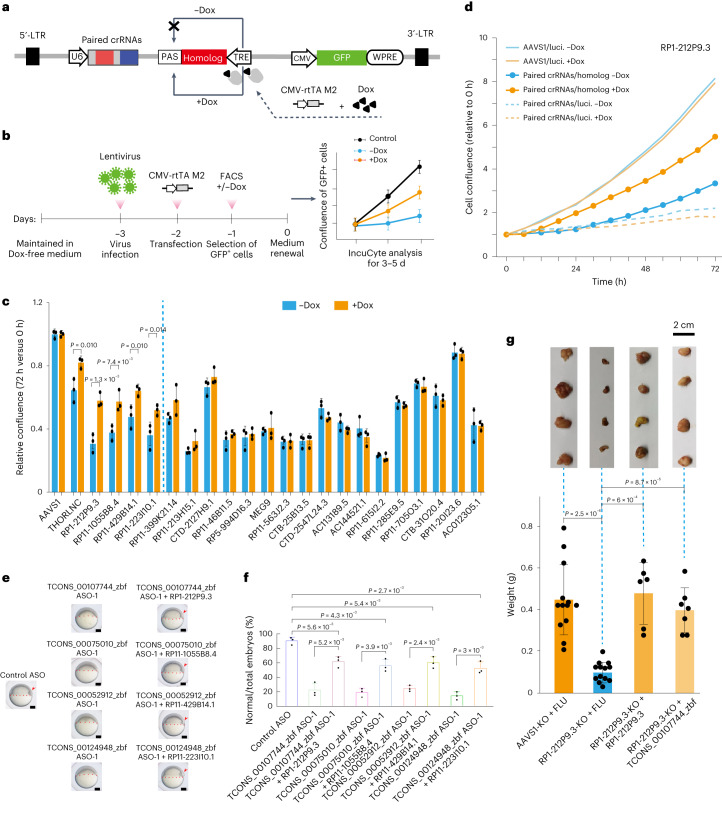
Functional validation of coPARSE-lncRNAs. **a**, KO-rescue lentivirus plasmid construction. The plasmid contains three functional cassettes for U6 promoter-driven expression of crRNAs, Dox-inducible ectopic expression of homologs and GFP labeling for infected cells. **b**, IncuCyte proliferation analysis. HeLa cells maintained in a Dox-free culture medium were split into two groups (Dox+/−) for lentivirus infection, followed by transient transfection of rtTA-expression or control pcDNA3.1 plasmids 24 h after infection. GFP-positive cells were sorted by FACS for IncuCyte proliferation analysis. Error bars, means ± s.d., *n* = 3 biologically independent experiments. **c**, KO-rescue assays of 21 candidate coPARSE-lncRNAs (THORLNC as a positive control). The relative cell confluence upon Dox induction was calculated for these coPARSE-lncRNAs (the fold change of 72 h versus 0 h for each coPARSE-lncRNA was normalized to AAVS1 in the Dox+/− groups). An AAVS1-targeting crRNA pair and a segment of fly luciferase gene were used for the AAVS1 group. Error bars, means ± s.d., *n* = 3 biologically independent experiments, two-sided Student’s *t*-test. **d**, IncuCyte assay of the human coPARSE-lncRNA RP1-212P9.3 and its zebrafish homolog TCONS_00107744_zbf, using luciferase segments as a negative control, *n* = 2 biologically independent experiments. **e**, Time-matched images of early embryogenesis showing that injection of the four human coPARSE-lncRNAs rescued the developmental defect of the corresponding zebrafish lncRNA homolog knockdown embryos. The epiboly edge is marked by red dotted lines, and the embryonic shield is indicated by red arrowheads. Scale bars, 100 μm. **f**, Quantification of zebrafish lncRNA knockdown embryos complemented with human homologous coPARSE-lncRNAs, showing a rescue of the developmental delay. *n* = 3 biologically independent experiments. The number of embryos in each injection group is detailed in Methods. Error bars, means ± s.d., two-sided Student’s *t*-test. **g**, HeLa cell line xenograft tumors of Dox+/− groups of the human lncRNA RP1-212P9.3 KO and complementation samples by RP1-212P9.3 and its zebrafish homolog (TCONS_00107744_zbf), showing increased tumor growth of the complementation samples (top). Bar plot showing tumor weights (bottom). Error bars, means ± s.d., *n* = 13, 14, 6 and 7 biologically independent experiments, one-sided Student’s *t*-test.

We next explored the functional conservation of coPARSE-lncRNAs using a CRISPR–Cas12a KO-rescue system, in which KO human lncRNAs were complemented with their predicted zebrafish homologs (Fig. 4a,b; Methods). After successfully testing doxycycline (Dox)-induced ectopic gene expression (Extended Data Fig. 7h,i), we transfected the Cas12a-expressing cancer cells using lentivirus particles targeting 21 human lncRNAs with rescue sequences of their zebrafish homologs (Fig. 4b, Extended Data Fig. 7j and Supplementary Table 8).

Proliferation assays revealed that all selected coPARSE-lncRNAs, except RP11-20I23.6, showed 30–70% decrease in the proliferation rate for the no-Dox group as compared to the control (Fig. 4c). The cells grown in Dox-containing media had increased proliferation rates compared to the no-Dox group for five coPARSE-lncRNA and homolog pairs, indicating functional compensation by these five zebrafish homologs to promote proliferation. Note that the overall sequence identity of the four pairs (excluding THORLNC) was quite low, ranging from 39.4% to 44.9% (Supplementary Table 9).

We next focused on the coPARSE-lncRNA RP1-212P9.3 as an example. The ectopic expression of the predicted zebrafish homologous region partially rescued the cell proliferation defect resulting from RP1-212P9.3 KO, whereas the expression of a firefly luciferase gene fragment of matched length conferred no rescue effect (Fig. 4d, Extended Data Fig. 7k,l and Supplementary Table 9).

**Extended Data Fig. 8:**
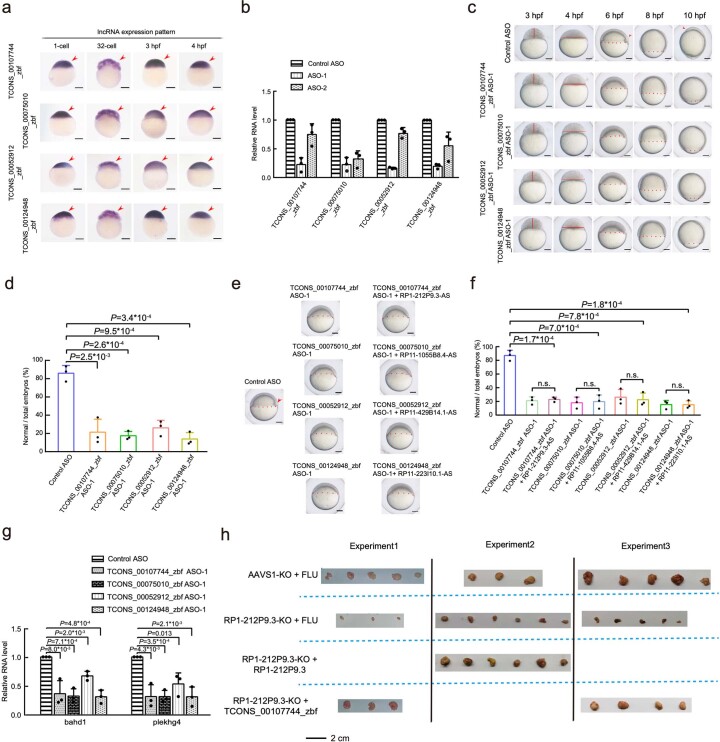
Validation of functional homology of RP1-212P9.3 and its zebrafish homolog in zebrafish early embryos and xenograft tumors. **a**, Expression pattern of four zebrafish lncRNA homologs of human coPARSE-lncRNAs in early zebrafish embryos. The expression pattern of the four zebrafish lncRNAs was analyzed using whole-mount in situ hybridization. Scale bar, 100 μm. **b**, Examination of ASO knockdown efficiency. The RNA levels of the four zebrafish lncRNAs knocked down by ASOs (two ASOs per lncRNA) were examined by RT-qPCR at 4 hpf. Data were normalized to *gapdh* then to control ASO knockdown embryos. n = 3 biologically independent experiments with 90 embryos, error bars, means ± SD. **c**, Time-matched images of early embryogenesis showed a developmental delay beginning at 4 hpf and continuing throughout subsequent gastrulation in the knockdown embryos. **d**, Quantification of developmental phenotypes. n = 3 biologically independent experiments with embryos (158 for control ASO, 134 for TCONS_00107744_zbf ASO-1, 152 for TCONS_00075010_zbf ASO-1, 74 for TCONS_00052912_zbf ASO-1, 133 for TCONS_00124948_zbf ASO-1), error bars, means ± SD, two-sided Student’s *t*-test. **e**, Time-matched images of early embryogenesis showing that injection of an antisense RNA for human coPARSE-lncRNAs did not rescue the developmental delay in corresponding zebrafish lncRNA homolog knockdown embryos at 6 hpf. For panels c and e, the height and width of the blastula are denoted by straight red lines. The epiboly edge is marked by dotted lines. The embryonic shield and polster are indicated by red arrowheads. Scale bars, 100 μm. **f**, Quantification of zebrafish lncRNA knockdown embryos complemented with antisense fragments of the human homologous coPARSE-lncRNAs showing no resue of developmental delay defect(s). n = 3 biologically independent experiments. The number of embryos in each injection groups is detailed in Methods. Error bars, mean ± SD, two-sided Student’s *t*-test, n.s., not significant. **g**, Down-regulation of zygotic genes in zebrafish lncRNA knockdown embryos. The relative mRNA levels of zygotic genes bahd1 and plekhg4 were examined by RT-qPCR in the control and zebrafish lncRNA knockdown embryos at 4 hpf. n = 3 biologically independent experiments with 90 embryos. Scale bars, 100 μm. Error bars, mean ± SD, two-sided Student’s *t*-test. **h**, Xenograft tumor mouse models of Dox+/− groups for the lncRNA RP1-212P9.3 or its homolog (TCONS_00107744_zbf) rescue samples.

We also assessed the potential functional conservation of predicted homologs for coPARSE-lncRNAs in early zebrafish embryo development. For the four coPARSE-lncRNAs identified in the rescue assay (RP1-212P9.3, RP11-1055B8.4, RP11-429B14.1 and RP11-223I10.1), we used antisense oligonucleotides (ASOs) to knockdown the predicted homologs in zebrafish early embryos^50–52^ and observed evident developmental delays as judged by morphologies^53^ (Fig. 4e,f and Extended Data Fig. 8a–d; Methods). Notably, the sense but not the antisense sequence of human coPARSE-lncRNA homologs rescued the development delay (Fig. 4e,f and Extended Data Fig. 8e,f). In addition, we found that knocking down the zebrafish lncRNA homologs led to reduced expression of known zygotic genes^54^ in zebrafish embryos, suggesting a delay in the zygotic gene activation process (Extended Data Fig. 8g).

Finally, we focused on RP1-212P9.3 and examined its functional conservation in a xenograft tumor model in mice. RP1-212P9.3 KO cells formed substantially smaller tumors than control AAVS1 KO cells. Moreover, the expression of human or zebrafish RP1-212P9.3 but notsubstantial the firefly luciferase gene fragment in the RP1-212P9.3 KO cells restored the tumor growth (Fig. 4g and Extended Data Fig. 8h). Together, these results support that coPARSE-lncRNAs have common regulatory impacts in distantly related species.

### Large overlap between coPARSE-lncRNAs homolog interactomes

**Extended Data Fig. 9:**
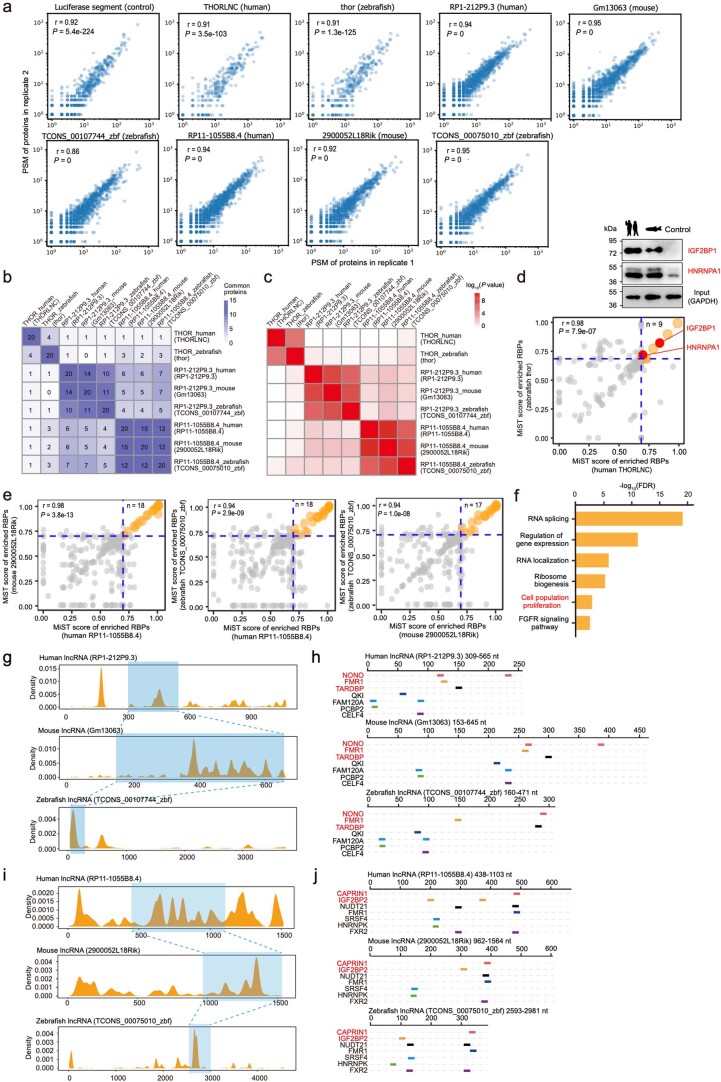
Identification and GO analysis of RBP interactome of two coPARSE-lncRNAs in human HeLa cells. **a**, Scatter plot showing the correlation between two biological replicates of peptide spectral matches (PSMs) of proteins pull-down by the candidate coPARSE-lncRNA segments with predicted homologs across species in HeLa cells. **b**, Heatmap showing the number of common proteins pulled-down by different lncRNAs. Top 20 pull-down proteins (top interactors) with highest MiST scores were analyzed. **c**, Heatmap showing the enrichment *P* value (two-sided Fisher’s exact test) of proteins pulled-down by different lncRNAs. **d**,**e**, Distribution of the MiST scores of pull-down RBPs by lncRNAs for (**d**) human THORLNC and its predicted homolog (thor) in zebrafish. Two commonly enriched RBPs from comparisons (highlighted in red circles) were validated by immunoblotting. (**e**) Human RP11-1055B8.4 and its predicted homologs (2900052L18Rik and TCONS_00075010_zbf) in mouse and zebrafish. The dashed lines represent a threshold of 0.7. For panels a, d, and e, r, Pearson correlation coefficient, two-sided Student’s *t*-test. **f**, The enrichment Gene Ontology (GO) terms related to the interacting proteins are shown. *P* values were calculated by two sided Fisher’s exact test and adjusted by FDR. **g**, Distribution of motif matches of RBPs in the human coPARSE-lncRNA RP1-212P9.3 and its homologs in mouse (Gm13063) and zebrafish (TCONS_00107744_zbf). Homologous regions are shadowed in blue. **h**, Distribution of motif matches of RBPs in homologous regions of human coPARSE lncRNA (RP1-212P9.3) and its homologs in mouse (Gm13063) and zebrafish (TCONS_00107744_zbf). **i**, Distribution of motif matches of RBPs in the human coPARSE-lncRNA RP11-1055B8.4 and its homologs in mouse (2900052L18Rik) and zebrafish (TCONS_00075010_zbf). Homologous regions are shadowed in blue. **j**, Distribution of motif matches of RBPs in homologous regions of human coPARSE lncRNA (RP11-1055B8.4) and its homologs in mouse (2900052L18Rik) and zebrafish (TCONS_00075010_zbf). For panels h and j, the motif matches for 7 representative RBPs with good alignment between three lncRNAs are shown. The RBPs predicted by lncHOME and identified by the RNA pulldown experiments in HeLa cell lysates are highlighted in red. Source data

We next tested if coPARSE-lncRNA homolog pairs interact with the same RBPs. We conducted RNA pull-down followed by mass spectrometry (MS) analysis for RP1-212P9.3 and RP11-1055B8.4 to examine interaction proteins of the human, mouse and zebrafish lncRNA homologs with HeLa cell lysates. Our MS data were of high quality (that is, correlation coefficients between biological replicates >0.85) and successfully recovered the interaction between THORLNC and IGF2BP1 (ref. ^21^; Extended Data Fig. 9a and Supplementary Table 10).

**Fig. 5:**
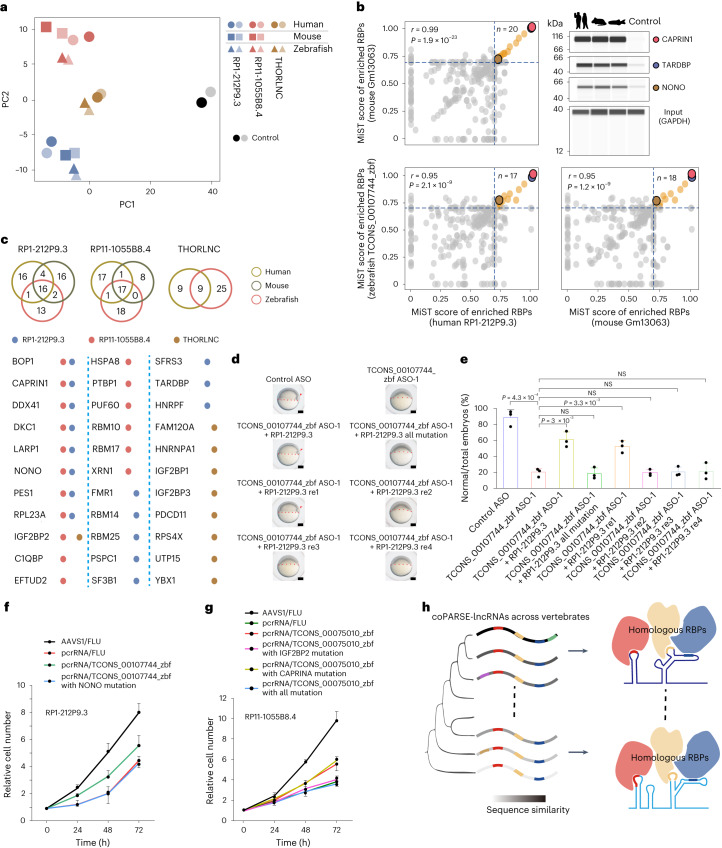
Identification and functional analysis of the RBP interactome for two coPARSE-lncRNAs. **a**, PCA of MS data for HeLa cell lysates pulled down for the indicated human coPARSE-lncRNAs and the predicted mouse and zebrafish homologs. The control samples are based on luciferase transcript segments. **b**, Distribution of the MiST scores of enriched RBPs upon pull-down using the human coPARSE-lncRNA RP1-212P9.3 and its predicted mouse and zebrafish homologs. Dashed lines represent a threshold of 0.7. Three commonly enriched RBPs from all comparisons (highlighted in colored circles) were validated by immunoblotting. The *r* represents the Pearson correlation coefficient, two-sided Student’s *t*-test. **c**, Venn diagram showing identified binding proteins of eight lncRNAs (human coPARSE-lncRNAs THORLNC, RP1-212P9.3 and RP11-1055B8.4, and their mouse and zebrafish homologs) in the RNA pull-down experiments (top). The table presents common binding proteins of three human lncRNAs and their homologs (bottom). Each dot represents a binding protein. **d**,**e**, Time-matched images (**d**) and quantifications (**e**) of early embryogenesis showing that injection of a human homologous coPARSE-lncRNA RP1-212P9.3 fragment and an RP1-212P9.3 fragment with the intact NONO-binding sites (RP1-212P9.3 re1) rescued the developmental defect of the corresponding zebrafish lncRNA homolog knockdown embryos. The epiboly edge is marked by red dotted lines, and the embryonic shield is indicated by red arrowheads. *n* = 3 biologically independent experiments. The number of embryos in each injection group is detailed in Methods. Scale bars, 100 μm. Error bars, means ± s.d., two-sided Student’s *t*-test. **f**,**g**, High-content imaging proliferation assays of RP1-212P9.3 (**f**) and RP11-1055B8.4 (**g**) KO HeLa cells rescued with wild-type zebrafish homologs and mutants bearing mutated RBP-binding sites. A luciferase segment was used as a negative control. AAVS1/FLU, control with pcrRNA targeting AAVS1 gene and overexpression of the luciferase segment. All groups were cultured with 500 ng ml^−1^ Dox. Error bars, means ± s.d., *n* = 3 biologically independent experiments. **h**, A simplified model for the evolution and function of coPARSE-lncRNAs. NS, not significant. Source data

Principal component analysis (PCA) of the pull-down proteins revealed that coPARSE-lncRNA homolog pairs are closer to each other than distinct lncRNAs in the same species in the embedding, strongly supporting the similarity of the binding protein profiles between coPARSE-lncRNA homolog pairs (Fig. 5a). We observed a very high correlation and extensive overlap for the enriched RBPs (MiST scores >0.7) and top interactors of coPARSE-lncRNA homolog pairs (Fig. 5b,c and Extended Data Fig. 9b–e). Immunoblotting confirmed that human coPARSE-lncRNA RP1-212P9.3 and its mouse and zebrafish homologs all interact with CAPRIN1, TARDBP and NONO (Fig. 5b). Gene Ontology (GO) analysis indicated that proteins interacting with the examined lncRNAs are enriched for cell proliferation-related functions (Extended Data Fig. 9f). The RNA pull-down experiments identified 6 and 5 RBPs in our RBP library used for motif-pattern analysis to bind RP1-212P9.3 and RP11-1055B8.4. It bears mention that 3 of 6 and 2 of 5 identified RBPs were accurately predicted by lncHOME for RP1-212P9.3 and RP11-1055B8.4, and there was good alignment of the motif matches between the coPARSE-lncRNA homolog pairs (Extended Data Fig. 9g–j).

**Extended Data Fig. 10:**
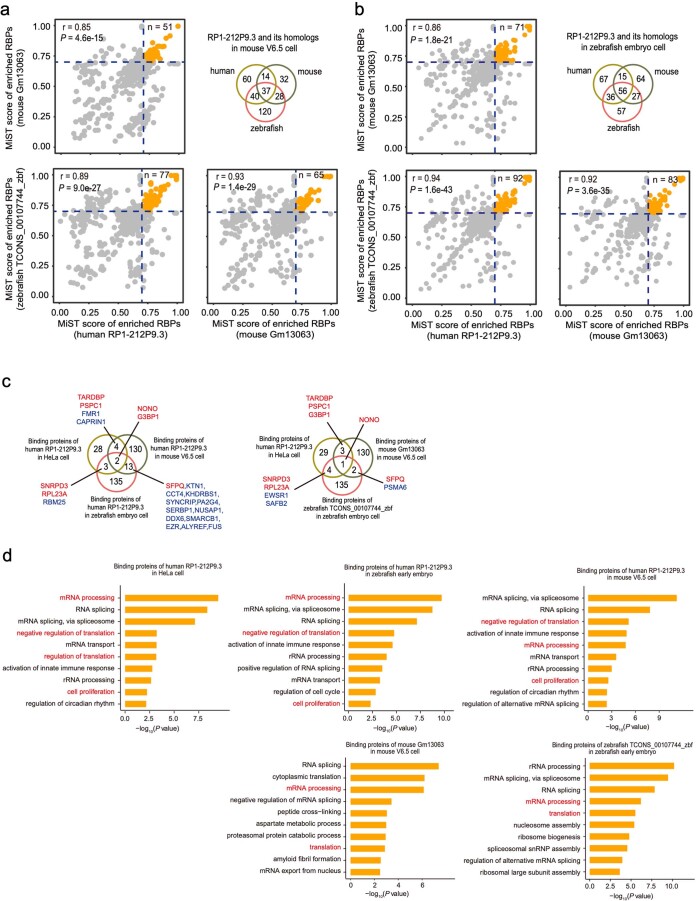
Identification and GO analysis of the RBP interactome of two coPARSE-lncRNAs in mouse cells and zebrafish embryos. **a**, Distribution of the MiST scores of enriched RBPs pulled down using the human coPARSE-lncRNA RP1-212P9.3 and its predicted homologs from mouse and zebrafish in mouse V6.5 cells. The dashed lines represent a threshold of 0.7. The yellow circles represent the enriched RBPs in both two lncRNAs. Venn diagram showing the overlap of identified binding proteins for RP1-212P9.3 and its predicted homologs in mouse V6.5 cells. R, Pearson correlation coefficient, two-sided Student’s *t*-test. **b**, Distribution of the MiST scores of enriched RBPs pulled down using the human coPARSE-lncRNA RP1-212P9.3 and its predicted homologs from mouse and zebrafish in zebrafish embryos. The dashed lines represent a threshold of 0.7. The yellow circles represent the enriched RBPs in both two lncRNAs. Venn diagram showing the overlap of identified binding proteins for RP1-212P9.3 and its predicted homologs in zebrafish embryos. R, Pearson correlation coefficient, two-sided Student’s *t*-test. **c**, Venn diagram showing the overlap of identified binding proteins for RP1-212P9.3 in HeLa cells, mouse V6.5 cells, and zebrafish embryos (left) and identified binding proteins for RP1-212P9.3 in HeLa cell, for its predicted mouse homolog in mouse V6.5 cells, and for its predicted zebrafish homolog in zebrafish embryos (right). Note that during the analysis, we only retained the proteins which have a homolog in all three species. The overlap proteins between the left and the right diagrams are highlighted in red. **d**, Enriched Gene Ontology (GO) terms related to the interacting proteins of human coPARSE-lncRNA RP1-212P9.3 and its predicted mouse and zebrafish homologs in human HeLa cells, mouse V6.5 cells, and zebrafish early embryos. *P* values were calculated by two-sided Fisher’s exact test.

We also conducted RNA pull-down and MS analyses for RP1-212P9.3 homologs in mouse cells (V6.5 embryonic stem cells) and early zebrafish embryos (Methods). Our results revealed strong correlation and extensive overlap in the enriched RBPs (MiST scores >0.7) for RP1-212P9.3 homologs in human HeLa cells, mouse V6.5 cells and zebrafish embryos (Fig. 5b and Extended Data Fig. 10a,b).

Additionally, we assessed the common proteins pulled down by RP1-212P9.3 homologs in the cells of the corresponding species. Because these cells are not from equivalent tissues and express drastically different sets of RBPs, we defined benchmark sets of common proteins that were pulled down by the same lncRNA RP1-212P9.3 in samples of different cell types. We noted a relatively high overlap between the pulled-down proteins by RP1-212P9.3 and its homologs in comparisons to the benchmark (Extended Data Fig. 10c). We also observed that the proteins pulled down by RP1-212P9.3 homologs showed enrichment for functions related to translation and cell proliferation (Extended Data Fig. 10d).

We next performed five additional complementation assays attempting to rescue the zebrafish early development delay defect resulting from TCONS_00107744_zbf knockdown, with each assay using a fragment of the predicted human homolog RP1-212P9.3 harboring distinct sets of putative RBP-binding sites (Methods). We found that only the fragment with intact NONO-binding sites rescued the developmental delay of the TCONS_00107744_zbf knockdown zebrafish embryos (Fig. 5d,e). These results demonstrate the specificity of the binding sites of an RBP (NONO) for the rescue fragments.

We also performed two KO-rescue experiments in HeLa cells for coPARSE-lncRNAs (RP1-212P9.3 and RP11-1055B8.4) and found that mutation of the NONO-binding site and the IGF2BP2-binding site, respectively, abolished the rescue effect of the TCONS_00107744_zbf fragment and the TCONS_00075010_zbf fragment (Fig. 5f,g). Together with the results from the zebrafish rescue experiments, these findings strongly suggest that coPARSE-lncRNA homologs are functionally related through interactions with specific RBPs.

## Discussion

We here developed lncHOME, a computational pipeline that identifies coPARSE-lncRNAs, a unique class of lncRNAs with conserved genomic locations and patterns of RBP-binding sites. We also developed a KO screen using Cas12a with paired crRNAs and identified 75 coPARSE-lncRNAs that functionally impact cancer cell proliferation. Moreover, several prioritized human coPARSE-lncRNAs and their zebrafish homologs were validated to exert common functions in distantly related species. Homologs of two coPARSE-lncRNAs from human, mouse and zebrafish share a large number of RBPs in their interactomes. Finally, experiments with mutant variants for particular RBP-binding sites established that specific RBP bindings impact the conserved functions (Fig. 5h). Our study thus provides a rich resource of conserved lncRNAs across vertebrates and sheds new light on the evolution of lncRNA functions.

Previous studies investigating lncRNA evolution have mostly relied on strategies developed to study protein-coding gene evolution, such as BLAST-like tools^19,20,24^ or UCSC LiftOver^55^. However, these protein sequence conservation analysis tools have achieved limited success for studying lncRNA evolution, and identifying lncRNA homologs across evolutionarily very divergent species has remained a formidable challenge. Unlike protein-coding genes, which are subjected to strong evolutionary pressure to maintain their primary sequences of open reading frames and codon synonyms, lncRNAs function through interacting with other biomolecules including DNA, RNA and proteins^3,56^.

It has been proposed that conserved functions of lncRNAs across different species may require only short specific sequences^24^. Notably, lncRNAs with similar *k*-mer content have been associated with similar regulatory roles in transcriptional regulation^57^. SEEKER^57^, a computational method based on lncRNA *k*-mer profiles, has been developed to identify groups of lncRNAs with similar functions. However, it is important to note that SEEKER is not designed to discriminate homologous lncRNAs within a functional group. In contrast, lncHOME achieves this goal by analyzing conserved genomic locations and distribution patterns of sequence motifs. Additionally, lncHOME uses motifs derived from known and validated RBP-binding sites, enabling our approach to generate testable hypotheses regarding the functions of coPARSE-lncRNAs.

In the present analysis, we used binding motifs of ~200 RBPs. However, the total number of known RBPs in humans is estimated to be around 2,000 (refs. ^30,58^). The list of RBP-binding motifs is expected to expand substantially as more transcriptome-wide profiling data for RBP-binding sites become available^41,59^. Future development of lncHOME may include the incorporation of other functional elements such as microRNA-binding sites. It is also worth noting that although our curated lncRNAs display extensive overlap with existing annotations, improved annotations in the future are likely to enhance the identification of coPARSE-lncRNAs. Consequently, the set of conserved lncRNAs is likely to expand. It should be interesting to search for homologs of coPARSE-lncRNAs between humans and evolutionarily distant species beyond vertebrates. The identification and study of these coPARSE-lncRNAs could provide insights into their fundamental biological roles and potentially shed light on the origin of lncRNAs.

Our coPARSE-lncRNA KO screening method takes advantage of the capability of Cas12a in processing paired crRNAs expressed as a single transcript under a U6 promoter. This approach minimizes the gRNA library construction procedure and prevents incorrect paired gRNA assembly caused by the recombination of two separate U6 promoter sequences used in the Cas9 approach^47^. Our method is applicable for dissecting the functions of protein-coding genes and noncoding elements, including promoters and enhancers, where genome deletions are preferred over mutations. While this study focused on cell proliferation, it is feasible to screen for coPARSE-lncRNAs essential in other cellular processes using suitable reporter systems, such as Nanog-GFP^60^ or miRNA activity reporters for cell differentiation^61^. Exploring coPARSE-lncRNA functions in different cellular processes is expected to expand the repertoire of known functionally conserved lncRNAs.

Our single-step KO-rescue approach illustrates an effective screening system for assessing the functional conservation of lncRNA homolog pairs from distantly related species. However, our current design involves ectopic expression of a fragment of the lncRNA that covers a lncHOME-predicted homologous region, instead of the full-length lncRNA. This design may cause an underestimation of the number of homologous lncRNAs with conserved functions, as other parts of the lncRNAs could contain motifs that are required for their (conserved) function. Moreover, it is worth noting that the overexpression levels of lncRNA fragments were not tightly controlled, and different cell types were used across species. These limitations may introduce potential artifacts into our interpretations, so it will be beneficial to develop assays that specifically address these constraints, especially in the context of high-throughput analysis involving a large number of coPARSE-lncRNAs.

## Methods

### Ethics statement

This research complies with all relevant ethical regulations. All animal protocols were approved by the Institutional Animal Care and Use Committees of Peking University, which are accredited by the Association for Assessment and Accreditation of Laboratory Animal Care International. All zebrafish experiments were approved and carried out in accordance with the Animal Care Committee at the Institute of Zoology, Chinese Academy of Sciences.

### Cell culture and reagents

HEK293T and HeLa cell lines were obtained from ATCC, and Huh7 and MCF7 cell lines were from the National Biomedical Cell Resource (Beijing). All cell lines were cultured in Dulbecco’s modified Eagle’s medium (Gibco) supplemented with 10% FBS (Dox-free, BI) at 37 °C. Mycoplasma kit (Vazyme) was used to routinely check for mycoplasma contamination in culture. For lncRNA rescue, Dox (Selleck) was added at a final concentration of 500 ng ml^−1^. The ZEM-2S zebrafish cell line was obtained from the China Center for Type Culture Collection and was cultured in 50% Leibovitz’s L-15 medium (Gibco), 35% DEEM (Gibco) and 15% F12 medium (Gibco) supplemented with 10% FBS (Hyclone) at 28 °C.

### Plasmid construction

The crRNA-expressing vector for genome deletion was constructed by cloning two tandem crRNAs (paired crRNAs) to downstream of the human U6 promoter of the lentiviral vector psWLV (a lentivirus plasmid, Addgene). For homolog rescue, an inducible expression cassette containing tetracycline-responsive element (TRE) promoter, homologous segments and bovine growth hormone (BGH)-polyA was inserted downstream of the cPPT site in a reverse transcription direction. The construction was done using a Gibson assembly strategy (TransGen Biotech) according to the manufacturer’s instructions.

### Construction of the cell line stably expressing Cas12a nucleases

A lentiviral vector expressing Cas12a-T2A-mCherry (Addgene) was packaged in 293T, and infection was performed in HeLa, Huh7 and MCF7. To obtain clones with high Cas12a expression, mCherry-positive cells were sorted as single cells into 96-well plates. After culturing for 3 weeks, the selected cell lines were tested for KO efficiency and those with the strongest KO effects were retained for further analysis.

### Construction of paired crRNA KO library

The CRISPR KO protocol was modified from that of the previous reports^62,63^. We created a library containing 11,301 pairs of crRNAs targeting 574 lncRNAs (including 249 coPARSE-lncRNAs with predicted zebrafish homologs) and 23 positive controls collected from previous studies (Supplementary Table 6). An additional 100 paired crRNAs were designed to target the introns of AAVS1 loci as the negative control. The 126-nt oligonucleotides containing pairs of tandemly arranged direct repeat sequences (19 nt) followed by a guide sequence (23 nt) with flanking adapters were synthesized by CustomArray. A pair of primers targeting the flanking adaptors was used for the PCR amplification of crRNA libraries with reaction systems in 24 tubes and at most 26 cycles. The amplified DNA products were ligated, using a Gibson cloning kit (TransGen Biotech), into a lentiviral vector linearized by BsmBI. The resulting assembly products were transformed into trans-T1 competent cells (TransGen Biotech) to obtain the plasmid library.

All colonies from transformation were reseeded in 16 flasks of 200 ml Luria broth liquid medium and cultured to the early exponential phase. The library plasmids were extracted using the EndoFree Plasmid Extraction Kit (CWbio). The lentivirus of the paired crRNA library was produced by cotransfecting library plasmids and packaging plasmids psPAX2 and pMD2.G (Addgene) into HEK293T cells using the jetPRIME DNA transfection reagent (Polyplus-transfection).

### CRISPR–Cas12a screening

Initial cell libraries were obtained through lentivirus infection at low multiplicity of infection (MOI; ~0.3), followed by sorting and collecting GFP-positive cells 72 h after infection using FACSAria II (BD Biosciences). For each sample, 2 million GFP-positive cells (~175-fold of the paired crRNA library size) were plated onto a 150-mm dish. Three replicate samples were processed for library screening, and one sample was used for genomic DNA extraction as the control group (day 0). During the screening, samples containing at least 4 million cells were collected for genomic DNA extraction at three time points (days 15, 30 and 45) after splitting.

### Identification of candidate-paired crRNA sequences

The genomic DNA was isolated from around 4 million cells using the Genomic DNA Kit (TianGen Biotech), and 32 μg DNA was used as amplification templates in 16 independent PCR (50-μl reaction each). The fragments containing paired crRNAs were first amplified using Q5 High Fidelity Polymerase (NEB; Fig. 3a and Supplementary Table 11). In the second round of PCR, primers for sequencing purposes with different indexes were added for different samples (replicates and time points). Finally, the PCR products of all samples were pooled and purified with a DNA Clean & Concentrator-5 Kit (Zymo Research) and sequenced by Illumina HiSeq 2500.

### Cell proliferation assay

For the validation of individual coPARSE-lncRNAs, the percentage of GFP-positive cells was quantified by flow cytometry analysis at 72 h postinfection (day 0) and every 5 d. Cell viability was determined by normalizing data to day 0. All virus infection assays were performed in 24-well plates with triplicates. Flow cytometry and data analysis were performed by the LSRFortessa SORP system and FlowJo software (BD Biosciences). For proliferation assay in a pure KO population, 0.2 × 10^4^ GFP-positive cells were seeded in triplicates in 96-well plates. Cell confluence (occupied area) was monitored by the IncuCyte ZOOM live-cell imaging system (Essen BioSciences, 2016a version). Data were normalized to time 0. Raw and processed statistical results are accessible in Supplementary Table 9.

### shRNA knockdown assay

The sequences of shRNAs were designed by an online tool (http://rnaidesigner.thermofisher.com/). The shRNA template was generated by overlap PCR from two short complementary oligonucleotide sequences with flanking primers and ligated into the lentiviral psWLV backbone through the Gibson assembly step. Scrambled shRNA was designed as a control. Lentivirus infection and cell proliferation analysis were performed as described above. Oligonucleotide sequences for constructing shRNAs were synthesized at Tsingke, and their sequences (in sense format) are listed in Supplementary Table 11.

### RNA isolation, cDNA synthesis and RT–qPCR

Total RNA was extracted using Trizol reagent (Invitrogen) according to the manufacturer’s instructions and further purified with an RNA Clean & Concentrator-5 Kit (Zymo Research). cDNAs were synthesized using random primers by PrimeScript RT Reagent Kit (Takara). RT–qPCR was performed with SYBR TB Green Premix (Takara) on an ABI qPCR system. The Actin was used as a control. RT–qPCR primers are shown in Supplementary Table 11.

### Cloning of cDNA for coPARSE-lncRNA homologs

cDNA for the predicted zebrafish coPARSE-lncRNA homologs were amplified from zebrafish mixture cDNA samples of different developmental phases (gift from A.M. Meng laboratory, Tsinghua University) or synthesized by TsingKe Biotech. For rescue plasmids, homologs amplified by PCR were inserted into AvrII (NEB, R0174S) digested rescue plasmids under the control of a Dox-inducible promoter using a Gibson Assembly Kit (NEB, E2611S). The sequences with adaptors for coPARSE-lncRNA homologs are listed in Supplementary Table 11.

### DNA isolation and genotyping PCR

For genomic DNA quick extraction, around 2,000 cells were lysed in 19 μl lysis buffer (10 mM Tris–HCl (pH 8.0), 2 mM EDTA and 0.2% Triton). After a freeze-thaw cycle under −80 °C, 1 μl proteinase K (10 mg ml^−1^) was added and the mixture was incubated at 55 °C for 2 h before heating at 95 °C for 10 min. Then, 1 μl of lysate was used directly for PCR genotyping. All genotyping primers are listed in Supplementary Table 11.

### Single-step CRISPR–Cas12a KO-rescue assay

To obtain high efficiency of lentivirus package and/or infection^64^, we tested multiple versions of construction and selected a highly efficient version in which the rescue cassette was inserted in a reverse transcription direction between two long terminal repeats (LTRs) of the lentiviral vector (Fig. 4a and Extended Data Fig. 7h). For rescue assay, HeLa cells stably expressing Cas12a-TA-mCherry were split into two groups (Dox+/−) during lentivirus infection and transfected with an rtTA-expression vector the day after infection. GFP-positive cells were then collected by FACS and split into 96-well plates the following day. The plates were loaded for IncuCyte proliferation analysis after culturing for 3 d.

### Design of rescue RNA fragments with RBP-binding sites mutated

Mutation of RBP-binding sites was made by replacing the original sequence with its antisense sequence. For the rescue of TCONS_00107744_zbf knockdown zebrafish embryos, we used fragments of the predicted human homolog RP1-212P9.3 harboring distinct sets of the putative RBP-binding sites. Especially, there are four RBPs (NONO, SF3A3, RBM22 and HNRNPC) (1) with predicted motif matches in both human and zebrafish homologs and (2) that were pulled down from zebrafish embryo lysates. We, therefore, designed the following five mutation fragments—(1) the sequence with all binding sites of the four RBPs mutated, (2–5) based on (1), but restoring the sequences at the binding sites for each of the four RBPs.

For rescue experiments in HeLa cells, we used fragments of the predicted zebrafish homologs with wild-type or mutated putative RBP-binding sites. For RP1-212P9.3, we designed a fragment of the predicted zebrafish homolog TCONS_00107744_zbf with the putative NONO-binding sites mutated. For RP11-1055B8.4, there are two RBPs (IGF2BP2 and CAPRINA) (1) with predicted motif matches in both zebrafish and human homologs and (2) that were pulled down from HeLa cell lysates. We thus designed the following three mutation fragments: (1) the sequence with all binding sites of the two RBPs mutated, (2) and (3) based on (1), but restoring the sequences at the binding sites for each of the two RBPs.

### Zebrafish husbandry and microinjection

Zebrafish (AB strain) were raised in a circulating aquarium system at 28.5 °C under standard conditions. Adult zebrafish aged between 3 months and 1 year were used for natural mating and egg collection, and the one-cell stage embryos were collected for microinjection experiments. ASOs were synthesized by GenePharma, and 80 pg per embryo was injected. The sequences are listed in Supplementary Table 11. The qPCR primers used for knockdown efficiency examination are listed in Supplementary Table 11. For human lncRNA rescue experiments, coPARSE-lncRNA or antisense RNA was generated by in vitro transcription using SP6 or T7 RNA polymerase (Promega). In total, 40 pg RNA per embryo was injected. The number of embryos in each experiment group is listed in Supplementary Table 9.

### Whole-mount in situ hybridization

Whole-mount in situ hybridization was carried out using Digoxigenin-uridine-5′-triphosphate (Roche) labeled antisense RNA probes as previously reported^65^. RNA probe was transcribed with SP6 RNA polymerase (Promega). After hybridization, RNA probes were detected by alkaline phosphatase (AP)-conjugated anti-digoxigenin (DIG) antibody (Roche) using Benjamin Moore (BM) purple (Roche, 11093274910; 1:20) as the substrate.

### Morphological feature assessment of zebrafish embryos

The developmental characteristics were assessed by the photomicrographs of zebrafish embryos. For the analysis, we measured the height of the blastula at 3 hpf (normal: 140 μm < *n* < 200 μm), the width at 4 hpf (normal: 390 μm < *n* < 450 μm) and the degree of epiboly process from 6 hpf to 10 hpf (normal: embryonic shield appeared and 45% < percent-epiboly < 55% at 6 hpf; 70% < percent-epiboly < 80% at 8 hpf; polster appeared and percent-epiboly = 100% at 10 hpf). The embryos with parameters falling out of the abovementioned ranges were defined as abnormal.

### In vivo xenograft experiments

Male mice (NOD/SCID) aged 5–7 weeks were injected with 1 million HeLa cells with stable integration of RP1-212P9.3 KO-rescue cassettes along with a Matrigel scaffold (BD Biosciences) in the posterior dorsal flank region. We used 10 mg ml^−1^ sucrose in drinking water supplemented with or without Dox (2 mg ml^−1^) to feed the mice. Animals were killed and subcutaneous tumors were excised and weighed at day 31 postcell injection.

### RNA pull-down assay

The in vitro RNA pull-down assay was performed as described previously^66^. Briefly, 100 pmol purified biotinylated RNA of candidate coPARSE-lncRNAs or luciferase fragment control was refolded and incubated with the lysate from 20 million mammalian cells or 2,500 zebrafish embryos at 4 °C for 2 h. Prewashed Dynabeads MyOne Streptavidin C1 beads (Invitrogen) were then added to the mixture and incubated at 4 °C for 45 min. After a series of washing, pull-down proteins were eluted in 15 μl elution buffer (1% SDS, 50 mM Tris–HCl (pH 8.0) and 1 M NaCl) and were subjected for MS or western blotting analysis.

### MS

The protein samples were analyzed by 10% SDS–PAGE and visualized by Fast Silver Stain Kit (Beyotime) according to the manufacturer’s instructions. The proteins were recovered from the bands in two or three split fragments per lane and each fragment was independently subjected to further MS analysis (performed by Tsinghua University Phoenix Center using LTQ-Orbitrap Velos Mass Spectrometer). MS raw results and processed MiST results are presented in Supplementary Table 10.

### Western blot analysis

The quantity of RNA pull-down proteins was determined by western blotting analysis using the Jess fully automated system (Bio-Techne) following the suggested protocols (https://www.proteinsimple.com/technical_library.html). The 12–230 kDa Jess Separation Module was used, and 3 μl of each sample was loaded. The incubation time of the primary and secondary antibodies was 30 min. Antibody against glyceraldehyde-3-phosphate dehydrogenase (GAPDH; ab9485, 1:500) from Abcam, against TARDBP (10782-2-AP, 1:100), NONO (11058-1-AP, 1:100), CAPRIN1 (15112-1-AP, 1:100), IGF2BP1 (22803-1-AP, 1:100) and hnRNPA1 (11176-1-AP, 1:100) from Proteintech. The secondary antibody (ab6721, 1:2,000) was from Abcam. Details of the primary antibodies are listed in Supplementary Table 11.

### LncRNA curation

We used the GENCODE data for human (GENCODE v25) and mouse (GENCODE vM10) lncRNA annotations. For the other six vertebrates (cow, opossum, chicken, lizard, frog and zebrafish), we obtained RNA-seq data from the National Center for Biotechnology Information (NCBI) to assemble lncRNA transcripts using established protocols^67,68^. The process involved quality-control (FASTQC v0.12.1), low-quality base trimming (Trimmomatic v0.39)^69^, mapping to the reference genomes (from UCSC Browser) using STAR 2.4.2a^70^ with a TwoPass Mode (parameter: --sjdbFileChrStartEnd), transcript assembly (StringTie v2.1.5)^71^ and merging (Cufflink v2.2.1)^72^, and filtering by length (≥200 nt), expression level (FPKM > 0.5) and protein-coding potential (CPAT v3.0.0 (ref. ^32^), CPAT score >0.5).

Additionally, we collected previously curated lncRNA from Ensembl, NCBI, NONCODE^73^, DeepBase^74^ and the Ulitsky laboratory^24^. We analyzed the overlap scores to compare lncRNA annotations from different sources:

$${\rm{Overlap}}\; {\rm{score}}=0.5\times \left(\frac{m}{{n}_{1}}+\frac{m}{{n}_{2}}\right)$$

Here *n*_1_ and *n*_2_ are the numbers of lncRNAs from dataset 1 and dataset 2, and *m* is the number of common lncRNAs.

### Conservation of protein-coding genes and lncRNAs between two vertebrates

For protein-coding and lncRNA genes, we performed pairwise sequence alignment to identify homologous genes with a high sequence similarity using BLAST v2.12.0 bl2seq (*E* value < 10^−4^, hit length >50 nt, overall sequence identity >50%). We then calculated a Jaccard index as the proportion of homologous genes among all genes to represent gene conservation between two vertebrates:

$${\mathrm{Jaccard}}\; {\rm{index}}=\frac{n}{x+y-n}$$

Here *x* and *y* are the numbers of protein-coding (or lncRNA) genes in species 1 and 2, and *n* is the number of homologous protein-coding (or lncRNA) genes between species 1 and 2.

### Identification of syntenic lncRNA candidates

We identified syntenic lncRNA candidates in different vertebrates by combining information from protein-coding genes (using OrthoDB^75^) and genomic anchors from pairwise genome alignments (using the UCSC chain extension files, if exist, or built using an in-house pipeline following the UCSC protocol). We only kept protein-coding genes and genomic anchors with one-to-one correspondence.

We used a random forest model to identify syntenic lncRNA candidates among humans (lncRNA1) and other species (lncRNA2). Briefly, we counted nine numbers within 1 Mbps of flanking genomic regions of the two lncRNAs, including the numbers of genomic anchors in the upstream, downstream and both the upstream and downstream regions (*m*_1*u*_, *m*_2*u*_, *m*_1*d*_, *m*_2*d*_, *m*_1*f*_ and *m*_2*f*_) and the numbers of genomic anchors with correspondence in lncRNA1 and lncRNA2 also in the three regions (*m*_*u*_, *m*_*d*_ and *m*_*f*_). We then defined three proportion scores based on these nine numbers, for the three regions (Extended Data Fig. 3b). As one example, for the upstream region, the proportion score is defined as

$${{\mathrm{Proportion}}\; {\rm{score}}}_{u}=\frac{{m}_{u}}{{\mathrm{minimum}}({m}_{1u},{m}_{2u})}$$

Similar to genomic anchors, we also defined nine numbers and three proportion scores based on protein-coding genes for lncRNA1 and lncRNA2 (we used protein homology from Ensembl^33^ as for correspondence of protein-coding genes). We finally used the six proportion scores and the six numbers (*m*_*u*_, *m*_*d*_ and *m*_*f*_) of genomic anchors and homologous protein-coding genes as 12 features for the training of a random forest model.

To train the model, we used protein-coding genes with one-to-one homology between humans and other species as positive samples, and randomly selected gene pairs between the two species as negative samples.

### RBP-binding motifs analysis

We downloaded CLIP data for the two RBPs (ELAVL1 and HNRNPA1)^34,76–78^ and called their binding site motifs from the 1,000 top-ranking binding peaks using HOMER^79^:


$findMotifs.pl binding_site.fa fasta output_directory/ -fasta background.fa


Here binding_site.fa contains the sequences of the binding peaks and background.fa contains the sequences of 1,000 permutated regions on the same transcripts with no RBP binding.

### Construction of RBP-binding motif libraries

For human and mouse, we collected RBP-binding motifs from RNACOMPETE^38^, CISBP-RNA^38^, RBPDB^40^ and ATtRACT^41^. We also called RBP-binding motifs from three public CLIP-seq datasets (CLIPdb^80^, eCLIP^34^ and Starbase^81^), using MEME (v4.10.1)^82^:


$meme input_file -p 5 -nostatus -time 36000 -dna -revcomp -text -mod anr -nmotifs 5 -minw 5 -maxw 30 -maxsites 600 -maxsize 1000000 > motif_file


Here input_file contains the sequences of top-ranking 1,000 RBP-binding peaks, and motif_file contains a position weight matrix of called motifs.

Then, for each RBP, we combined the binding motifs from the database collection and the motifs from CLIP-seq data calling (using TOMTOM v5.5.4 (ref. ^83^), *P* < 0.001) to define the human and mouse RBP-binding motif libraries.

We extrapolated the established human and mouse motifs to obtain more RBP motifs for human and mouse and to define all motifs for the other six species. First, we downloaded the RBP domain annotation for 263 human RBPs from the UniProt^84^ and defined homologous RBPs (alignment coverage ≥70% and alignment identity ≥70%)^85^.

We then extrapolated the human motifs to the mouse or the mouse motifs to the human, using an iterative mapping-and-refinement strategy, using FIMO (v4.11.2) for motif match searching:


$fimo --verbosity 1 --text motif_file sequence_file > motif_match_file


Here sequence_file contains target sequences, and motif_match_file contains motif matches.

Then we defined a new motif by combining the old motif and the matched sequences. For each of the other six species, we extrapolated every human motif to define a corresponding new species-specific motif.

### Identification of coPARSE-lncRNAs

We identified homologous RNA from the syntenic lncRNA candidates between humans and the other seven species. Briefly, we first scanned for motif matches along the sequences of syntenic lncRNA candidates using the above-curated species-specific motif libraries by FIMO (v4.11.2):


$fimo --verbosity 1 --text motif_file sequence_file > motif_match_file


We clustered the motif matches with half of the motif matches overlapped with the other into one block. Then for a candidate pair of lncRNA homologs from any two species, we defined a similarity score for every pair of blocks from the lncRNA pair:

$${\mathrm{Block}}\; {\rm{similarity}}\; {\rm{score}}=\mathop{\sum }\limits_{i=1}^{n}\frac{\min ({x}_{i},{y}_{i})}{\max ({x}_{i},{y}_{i})}.$$

Here *x*_*i*_ and *y*_*i*_ are the numbers of matched motif sites of motif class *i* on the lncRNA from the two species, and *n* is the number of motif class.

We used a dynamic programming algorithm to calculate an MPSS, which was summed up by the block similarity scores based on the optimal alignment of all block pairs. We also calculated a GPS, defined as the quadratic mean of the distance deviation of all paired blocks.

$${\rm{Gap}}\; {\rm{penalty}}\; {\rm{score}}=\frac{\sqrt{\mathop{\sum }\limits_{i=1}^{n-1}{({x}_{i}-{y}_{i})}^{2}}}{{{n}}-1}$$

Here *x*_*i*_ and *y*_*i*_ are the block distance between two blocks in the two lncRNAs, and *n* is the number of blocks.

We then calculated two *P* values for each pair of the predicted lncRNA homologs, one for MPSS and one for GPS (permutation test by sampling 100,000 random lncRNA pairs from different species and by shuffling the block positions for 1,000 times). We defined all lncRNA pairs having both two *P* values smaller than 0.05 as ‘coPARSE-lncRNA’ candidates. For a human coPARSE-lncRNA with more than one homolog candidate in another species, we only retained the candidates having an MPSS >0.8 times of the maximum MPSS among all candidates.

We defined the homologous regions for any pair of homologous lncRNAs as the sequence regions between the first aligned motif match and the last aligned motif match based on the alignment of motif matches using dynamic programming (Extended Data Fig. 9g–j). These homologous regions were used for designing lncRNA fragments for rescue and RNA pull-down experiments (only one fragment was used for each coPARSE-lncRNA).

### Species conservation analysis of human coPARSE-lncRNAs

We defined the following two groups of coPARSE-lncRNA homolog pairs: (1) The ‘homolog_ss’ groups containing 605 coPARSE-lncRNA homolog pairs with sequence similarity between human and mouse and 17 coPARSE-lncRNA homolog pairs with sequence similarity between human and zebrafish; (2) the ‘homolog_nss’ groups containing 4,959 coPARSE-lncRNA homolog pairs without sequence similarity between human and mouse and 553 coPARSE-lncRNA homolog pairs without sequence similarity between human and zebrafish. We also defined a third ‘non_homolog’ group containing randomly selected lncRNA pairs.

We calculated the distribution of average conservation scores based on the PhastCon and PhyloP scores (from UCSC^42,43^) for human lncRNAs of these three groups and compared the distributions by calculating a *P* value for the significance of score differences using two-sided Mann–Whitney *U* tests.

### SNP enrichment analysis of human coPARSE-lncRNAs

To evaluate selection for coPARSE-lncRNAs, we analyzed the SNP density for human coPARSE-lncRNAs. We first separated each coPARSE-lncRNA sequence into motif and nonmotif regions, based on the lncHOME pipeline. We compared the density difference of common SNPs (major alternative allele frequency >5%, the 1000 Genomes Catalog^86^) and the difference of major alternative allele frequencies of SNPs between the motif and nonmotif regions, by calculating a *P* value using a two-sided Mann–Whitney *U* test.

### Histone modification analysis

We collected data for seven types of histone modifications (H3K27ac, H3K27me3, H3K36me3, H3K4me1, H3K4me3, H3K9ac and H3K9me3) from the ENCODE dataset^87^. We calculated the rate of common histone modification sites between each lncRNA pair.

$${\mathrm{Common}}\; {\rm{histone}}\; {\rm{modification}}\; {\rm{site}}\; {\rm{rate}}=\frac{\mathop{\sum }\limits_{i=1}^{n}\min ({x}_{i},{y}_{i})}{\mathop{\sum }\limits_{i=1}^{n}\max ({x}_{i},{y}_{i})}$$

Here *x*_*i*_ and *y*_*i*_ are the numbers of each type of histone modification sites in human (*x*_*i*_) and mouse (*y*_*i*_) lncRNA genes and nearby regions (10 kb upstream and downstream regions), and *n* is the number of histone modification types. We compared the common histone modification site rate between each pair of lncRNAs for the above-defined three groups (homolog_ss, homolog_nss and non_homolog), by calculating a *P* value using two-sided Mann–Whitney *U* test.

### Tissue-specific expression analysis

We compared the tissue-specific expression scores for the ‘homolog_ss’ and ‘homolog_nss’ groups of coPARSE-lncRNAs between each two species based on the gene expression data from the Genotype-Tissue Expression (GTEx) Portal^88^, by calculating the Pearson correlation coefficients. We randomly selected lncRNA pairs from the two species, with and without synteny, to calculate the average Pearson correlation coefficient.

### Enrichment analysis of ClinVar variations

We collected disease-associated variants from ClinVar^44^. We randomly selected lncRNAs (the same number as the human coPARSE-lncRNA set) from the whole transcriptome and counted the numbers of these random lncRNAs with ClinVar variants. We repeated this process for 100,000 times to construct a background distribution to estimate the *P* value. The enrichment of ClinVar variants in human coPARSE-lncRNAs was calculated as follows:

$${\mathrm{Enrichment}}=\frac{{\mathrm{number}}\; {\mathrm{of}}\; {\mathrm{human}}\; {\mathrm{coPARSE}}-{\mathrm{lncRNAs}}\; {\mathrm{with}}\; {\mathrm{ClinVar}}\; {\mathrm{variants}}\,}{{\mathrm{number}}\; {\mathrm{of}}\; {\mathrm{randomly}}\; {\mathrm{selected}}\; {\mathrm{lncRNAs}}\; {\mathrm{with}}\; {\mathrm{ClinVar}}\; {\mathrm{variants}}}$$

### Differential coPARSE-lncRNA expression analysis for cancer tissues

We calculated the differentially expressed genes between normal and disease tissues for different types of cancer. The enrichment (odds ratio) of human coPARSE-lncRNAs with predicted homologs in mouse (coPARSE-lncRNAs in the following formula) for differentially expressed lncRNAs in patients with cancer compared to lncRNAs without predicted homologs in mouse (nonhomologous lncRNAs in the following formula) was calculated as follows:

$${\mathrm{Odds}}\,{\mathrm{ratio}}=\frac{\frac{{\mathrm{number}}\,{\mathrm{of}}\,{\mathrm{coPARSE}}-{\mathrm{lncRNAs}}\,{\mathrm{differentially}}\,{\mathrm{expressed}}\,{\mathrm{in}}\,{\mathrm{cancer}}\,{\mathrm{patient}}}{{\mathrm{number}}\,{\mathrm{of}}\,{\mathrm{coPARSE}}-{\mathrm{lncRNAs}}\,{\mathrm{normally}}\,{\mathrm{expressed}}\,{\mathrm{in}}\,{\mathrm{cancer}}\,{\mathrm{patient}}}}{\frac{{\mathrm{number}}\,{\mathrm{of}}\,{\mathrm{nonhomologous}}\,{\mathrm{lncRNAs}}\,{\mathrm{differentially}}\,{\mathrm{expressed}}\,{\mathrm{in}}\,{\mathrm{cancer}}\,{\mathrm{patient}}}{{\mathrm{number}}\,{\mathrm{of}}\,{\mathrm{nonhomologous}}\,{\mathrm{lncRNAs}}\,{\mathrm{normally}}\,{\mathrm{expressed}}\,{\mathrm{in}}\,{\mathrm{cancer}}\,{\mathrm{patient}}}}$$

The *P* value of the enrichment was estimated using Fisher’s exact test.

### Selection of candidate lncRNAs for CRISPR–Cas12a KO screening

To select lncRNA candidates for KO screening, we defined a set of candidate lncRNAs (including coPARSE-lncRNAs) that show high expression levels in cancer. We started from 570 human coPARSE-lncRNAs with predicted homologs in zebrafish, 511 human lncRNAs with predicted syntenic lncRNA candidates in zebrafish and 252 human lncRNAs with zebrafish homologs from the ZFLNC database^89^. We selected those lncRNAs with widespread expressions across various cancer tissues and cell lines (data from GTEx^88^, TANRIC^90^ and CCLE^91^) and finally defined a list of 574 human lncRNAs (including 249 coPARSE-lncRNAs).

For positive controls, we included 4 protein-coding and 19 lncRNA genes with reported proliferation function (Supplementary Table 6). For negative controls, we used the nontargeting region AAVS1 introns, which are located in an open chromatin region, and insertion or deletion of this region leads to no known adverse effects on the cell.

### Paired crRNA design and filtering

When designing crRNA pairs for a particular lncRNA, we first obtained all crRNAs that can target this lncRNA by considering factors potentially impacting efficiency and specificity of crRNAs (for example, protospacer adjacent motif (PAM) sequence TTTV^92^, GC contents), following a strategy previously reported^47^. To avoid off-target bias and low cleavage efficiency, we followed the guidelines of the aforementioned study and only retained a crRNA if (1) its sequence was uniquely mapped to the intended loci, (2) having at least two mismatches to any other loci of the genome, (3) its GC content was between 0.2 and 0.9 and (4) the crRNA did not include a UUUU polymer.

We then enumerated all possible crRNA pairs and selected those based on the following conditions: (1) both crRNAs flanking the TSS of the target lncRNA, (2) neither of the two crRNAs targeting any exon of a coding gene and (3) both crRNAs targeting the nontranscribed strand (a strategy has been shown to have higher KO efficiency than targeting the transcribed strand^47,93^).

Additionally, we have tried to avoid crRNA pairs overlapping with 1,580 essential genes (defined by a high-resolution CRISPR screen in 3 of 5 cell lines^94^). In the end, only 56 of the 574 (or ~9.8%) target lncRNAs have crRNA pairs that overlap with an essential gene. Sequences of crRNA pairs are listed in Supplementary Table 6.

### Computational analysis of KO screening

The whole processing procedure includes reads preprocessing, reads mapping, normalization of the count table and enrichment analysis.

First, we trimmed the raw reads to remove flanking sequences of the crRNAs (cutadapt v1.18 (ref. ^95^), parameters: -m 60 -M 70 -g GCATTCGGTCCGTAGCCAAAA…TCTACAAGAGTAGAAATTCTTTCGTCCTTTC -e 0.2 --overlap 5 -q 30,30), and then sampled 8 million reads for each screening sample using vsearch (v2.23.0)^96^.

Second, we used Bowtie2 (v2.2.5) to map the clean reads to the reference library (parameters: --local --score-min C,95 -D 20 -R 2 -N 1 -L 20 -i S,1,0.75 --norc).

Third, we used MAGeCK (v0.5.9.5)^97^ to obtain read count tables from the mapping results. The count tables were further normalized using RUVseq^98^ to remove variations using the AAVS reads pool as a negative control. The normalized reads were finally used for enrichment analysis to obtain significantly depleted genes during the screening of the cell culture.

We adapted a time-serial polynomial modeling method and combined it with the RRA algorithm^99^ for enrichment analysis, based on the data of multiple time points. Specifically, we fit the time-serial data of all paired crRNAs with a cubic polynomial function using ‘nlme’ (https://svn.r-project.org/R-packages/trunk/nlme/). We then calculated the rankings for all paired crRNAs based on their changes across time relative to the background controls of AAVS-derived paired crRNAs. We input the paired crRNA rankings into the RRA algorithm to calculate candidate genes.

### Filtering based on CNV and protein-coding gene overlapping

We used the CNV data for HeLa and MCF7 cells from ENCODE. We calculated an enrichment score of all of 574 lncRNA candidates within these CNV regions.

### MS data analysis

Following an established protocol^100^, we analyzed the MS data files using Proteome Discoverer (v1.4), using human protein sequences from UniProt^84^. We defined valid proteins by applying a minimum protein score of 1.5. We performed intersample comparison using the MiST algorithm^100^ and scored all valid proteins with default parameters (MiST score >0.7).

For paired coPARSE-lncRNA homologs, we calculated the correlation coefficient of the MiST scores of their interacting proteins, to evaluate the similarity of the two interacting protein sets and calculated a *P* value by the chi-squared test.

### GO enrichment analysis

We performed GO enrichment analysis for interacting proteins of coPARSE-lncRNAs using STRING (v11)^101^. The significant *P* values of GO terms were calculated by Fisher’s exact test and adjusted by false discovery rate (FDR).

### Statistics and reproducibility

Statistical methods for all analyses are detailed in the corresponding Methods section. No statistical method was used to predetermine the sample size. No data were excluded from the analyses. In this study, the reported results were acquired using independent mouse and fish that were randomly collected for each group. The investigators were not blinded to allocation during experiments and outcome assessment. All codes to replicate the analysis are available as part of code availability. Statistical analysis and related plots were carried out using R packages or Python Jupyter Note. For Student’s *t*-test, data distribution was assumed to be normal but this was not formally tested.

### Reporting summary

Further information on research design is available in the Nature Portfolio Reporting Summary linked to this article.

## Online content

Any methods, additional references, Nature Portfolio reporting summaries, source data, extended data, supplementary information, acknowledgements, peer review information; details of author contributions and competing interests; and statements of data and code availability are available at 10.1038/s41588-023-01620-7.

### Supplementary information

Reporting Summary

Supplementary Tables 1 and 2 Identification of syntenic lncRNAs across species.

Supplementary Table 3 Curation of RNA motifs for eight vertebrates.

Supplementary Table 4 Identification of coPARSE-lncRNA homologs across vertebrates by lncHOME.

Supplementary Tables 5–11 Experimental data and results for coPARSE-lncRNA function and interactome.

### Source data

Source Data Fig. 5 Unprocessed scans of gels for Fig. 5b.

Source Data Extended Data Fig. 5 Unprocessed scans of gels for Extended Data Fig. 5c.

Source Data Extended Data Fig. 7 Unprocessed scans of gels for Extended Data Fig. 7d.

Source Data Extended Data Fig. 9 Unprocessed scans of gels for Extended Data Fig. 9d.

## Data Availability

The sequencing datasets have been deposited in the Gene Expression Omnibus under the accession code GSE240342. The MS proteomics data have been deposited to the ProteomeXchange Consortium via the PRIDE partner repository with the dataset identifier PXD046452. The RNA-seq data source is provided in Supplementary Table 1. All datasets used in this study are available in supplementary tables and https://github.com/huangwenze/lncHOME_analysis. Source data are provided with this paper.

## Code availability

All the codes used for computational prediction and data analysis are available at 10.5281/zenodo.10162676 (ref. ^102^) and https://github.com/huangwenze/lncHOME_analysis.
