# Supplementary material for: Computational prediction and experimental validation identify functionally conserved lncRNAs from zebrafish to human
Source: Nat Genet. 2024 Jan 9;56(1):124–35. doi: 10.1038/s41588-023-01620-7 (PMC10786727; doi:10.1038/s41588-023-01620-7)
Supplement: Supplementary file 1 — Reporting Summary [file 41588_2023_1620_MOESM1_ESM.pdf]

nature portfolio

Corresponding author(s): Jianzhong Jeff Xi, Yangming Wang, Qiangfeng Cliff Zhang

Last updated by author(s): Oct 30, 2023

Reporting Summary

Nature Portfolio wishes to improve the reproducibility of the work that we publish. This form provides structure for consistency and transparency in reporting. For further information on Nature Portfolio policies, see our Editorial Policies and the Editorial Policy Checklist.

Statistics

For all statistical analyses, confirm that the following items are present in the figure legend, table legend, main text, or Methods section.

n/a	Confirmed	
☐	☒	The exact sample size (*n*) for each experimental group/condition, given as a discrete number and unit of measurement
☐	☒	A statement on whether measurements were taken from distinct samples or whether the same sample was measured repeatedly
☐	☒	The statistical test(s) used AND whether they are one- or two-sided<br>*Only common tests should be described solely by name; describe more complex techniques in the Methods section.*
☐	☒	A description of all covariates tested
☐	☒	A description of any assumptions or corrections, such as tests of normality and adjustment for multiple comparisons
☐	☒	A full description of the statistical parameters including central tendency (e.g. means) or other basic estimates (e.g. regression coefficient) AND variation (e.g. standard deviation) or associated estimates of uncertainty (e.g. confidence intervals)
☐	☒	For null hypothesis testing, the test statistic (e.g. *F*, *t*, *r*) with confidence intervals, effect sizes, degrees of freedom and *P* value noted<br>*Give P values as exact values whenever suitable.*
☒	☐	For Bayesian analysis, information on the choice of priors and Markov chain Monte Carlo settings
☐	☒	For hierarchical and complex designs, identification of the appropriate level for tests and full reporting of outcomes
☐	☒	Estimates of effect sizes (e.g. Cohen's *d*, Pearson's *r*), indicating how they were calculated

Our web collection on statistics for biologists contains articles on many of the points above.

Software and code

Policy information about availability of computer code

Data collection	RNA-seq data was collected from the NCBI website. The lncRNA annotations were downloaded from the other sources (e.g., Ensembl, NCBI, DeepBase). For one-to-one homology of protein-coding genes, we used the OrthoDB database. For pairwise genome alignments, we either obtained the data from the UCSC database. RBP binding motifs were collected from several databases, including CISBP-RNA, RBPDB, ATtRACT, and RNACOMPETE. CLIP-seq data was collected from CLIPdb, eCLIP, and Starbase datasets. We downloaded species conservation scores from UCSC database, SNP from the 1000 Genomes Catalog, and disease-associated variants from ClinVar database. We collected data of histone modifications determined in human and mouse liver tissues from the ENCODE dataset and gene expression data of three species from the Genotype-Tissue Expression (GTEx) Portal. The sequencing datasets have been deposited in the Gene Expression Omnibus (GEO) under the accession code GSE240342. The mass spectrometry proteomics data have been deposited to the ProteomeXchange Consortium via the PRIDE partner repository with the dataset identifier PXD046452.

Data analysis	Raw reads of RNA-seq data were quality-controlled using FASTQC (v0.12.1), pre-processed using Trimmomatic (v0.39), and mapped to the reference genomes using STAR 2.4.2a. StringTie (v2.1.5) was used to assemble transcripts, and the Cufflink (v2.2.1) tool was used for transcripts merging. CPAT (v3.0.0) was used to estimate protein-coding potentials of the resulting transcripts. LiftOver (v1.1) was used to transformed the genomic coordinates to the latest formal versions of the UCSC Browser database. BLAST (v2.12.0) was used to perform pairwise sequence alignment. MEME suite (v4.10.1) and HOMER (only one version) was used to call RBP binding motifs from CLIP-seq datasets. TOMTOM (v5.5.4) was used to calculate motif similarity. FIMO (v4.11.2) was used to search for motif matched sites in transcripts. For KO screening analysis, RUVseq (v1.34.0) package was used to normalize read counts and vsearch (v2.23.0) was used to subsampled reads. MAGeCK (v0.5.9.5) was used to obtain read count tables for all samples from the SAM files of mapping results. For Mass spectrometry data analysis, Proteome Discoverer (v1.4) was used to identified proteins from mass spectrometry data and MiST algorithm was used to identified interacting proteins. STRING (v11) was used for GO enrichment analyses. All the code used for computational prediction and data analysis is available at https://github.com/lynhsiong/lncHOME and https://github.com/huangwenze/lncHOME_analysis.

For manuscripts utilizing custom algorithms or software that are central to the research but not yet described in published literature, software must be made available to editors and reviewers. We strongly encourage code deposition in a community repository (e.g. GitHub). See the Nature Portfolio guidelines for submitting code & software for further information.

Data

Policy information about availability of data

All manuscripts must include a data availability statement. This statement should provide the following information, where applicable:

- Accession codes, unique identifiers, or web links for publicly available datasets
- A description of any restrictions on data availability
- For clinical datasets or third party data, please ensure that the statement adheres to our policy

The sequencing datasets have been deposited in the Gene Expression Omnibus (GEO) under the accession code GSE240342. The mass spectrometry proteomics data have been deposited to the ProteomeXchange Consortium via the PRIDE partner repository with the dataset identifier PXD046452. The RNA-seq data source is provided in Supplementary Table 1. All datasets used in this study are available in supplementary tables and https://github.com/huangwenze/lncHOME_analysis.

Human research participants

Policy information about studies involving human research participants and Sex and Gender in Research.

Reporting on sex and gender	No human research participant
Population characteristics	No
Recruitment	No
Ethics oversight	No

Note that full information on the approval of the study protocol must also be provided in the manuscript.

Field-specific reporting

Please select the one below that is the best fit for your research. If you are not sure, read the appropriate sections before making your selection.

☒ Life sciences ☐ Behavioural & social sciences ☐ Ecological, evolutionary & environmental sciences

For a reference copy of the document with all sections, see nature.com/documents/nr-reporting-summary-flat.pdf

Life sciences study design

All studies must disclose on these points even when the disclosure is negative.

Sample size	No statistical method was used to predetermine sample size.
Data exclusions	No data were excluded from the analyses.
Replication	For RT-qPCR analysis, at least two biological independent samples were used. For CRISPR-Cas12a knockout screen, at least three biological independent samples were used. For CRISPR-Cas12a knockout-rescue assay, at least three biological independent samples were used. For the knockout-rescue experiments in HeLa cells and knockdown-rescue experiments in zebrafish early embryos, at least three biological independent samples were used. For the RNA pull-down and MS analyses, at least two biological independent samples were used.
Randomization	In this study, the reported results were acquired using independent mouse and fish that were randomly collected for each group.
Blinding	The investigators were not blinded to allocation during experiments and outcome assessment.

Reporting for specific materials, systems and methods

We require information from authors about some types of materials, experimental systems and methods used in many studies. Here, indicate whether each material, system or method listed is relevant to your study. If you are not sure if a list item applies to your research, read the appropriate section before selecting a response.

Materials & experimental systems

n/a	Involved in the study
☐	☒ Antibodies
☐	☒ Eukaryotic cell lines
☒	☐ Palaeontology and archaeology
☐	☒ Animals and other organisms
☒	☐ Clinical data
☒	☐ Dual use research of concern

Methods

n/a	Involved in the study
☒	☐ ChIP-seq
☐	☒ Flow cytometry
☒	☐ MRI-based neuroimaging

Antibodies

Antibodies used	For western blot, For western blot, anti-GAPDH (Abcam, ab9485, 1:500), anti-TARDBP (proteinTech, 10782-2-AP,1:100), anti-NONO (proteinTech, 11058-1-AP,1:100), anti-CAPRIN1 (proteinTech, 15112-1-AP, 1:100), anti- IGF2BP1 (proteinTech, 22803-1-AP, 1:100), hnRNPA1 (proteinTech, 11176-1-AP,1:100), and HRP-conjugated goat anti-rabbit (Abcam, ab6721, 1:2000)<br>For in situ Hhybridization, AP-conjugated anti-DIG antibody (Roche, 11093274910,1:20)was used.
Validation	The validation of commercially available antibody was available on the manufacturer's website<br>GAPDH: https://www.abcam.com/products/primary-antibodies/gapdh-antibody-loading-control-ab9485.html<br>TARDBP: https://www.ptgcn.com/products/TARDBP-Antibody-10782-2-AP.htm<br>NONO: https://www.ptgcn.com/products/NONO-Antibody-11058-1-AP.htm<br>CAPRIN1: https://www.ptgcn.com/products/CAPRIN1-Antibody-15112-1-AP.htm<br>IGF2BP1: https://www.ptgcn.com/Products/IGF2BP1-Antibody-22803-1-AP.htm<br>hnRNPA1: https://www.ptgcn.com/Products/HNRNPA1-Antibody-11176-1-AP.htm<br>Goat Anti-Rabbit IgG H&L (HRP): https://www.abcam.cn/products/secondary-antibodies/goat-rabbit-igg-hl-hrp-ab6721.html<br>AP-conjugated anti-DIG antibody: https://www.sigmaaldrich.cn/CN/zh/product/roche/11093274910

Eukaryotic cell lines

Policy information about cell lines and Sex and Gender in Research

Cell line source(s)	HEK293T (ATCC), HeLa (ATCC), Huh7 (BMCR), MCF7 (BMCR), ZEM-2S(CCTCC), V6.5 mouse ESC line was previously generated by us (Please see Wang Y.et al., Nature Genet 39,380-385 (2007))
Authentication	All cell lines were used as received without further authentication.
Mycoplasma contamination	All cell lines were negative for mycoplasma contaminatioin.
Commonly misidentified lines (See ICLAC register)	No commonly misidentified cell lines were used in the study.

Animals and other research organisms

Policy information about studies involving animals; ARRIVE guidelines recommended for reporting animal research, and Sex and Gender in Research

Laboratory animals	Male mice (NOD/SCID, 5-7 weeks), Zebrafish (AB strain, aged between 3 months to one year)
Wild animals	The study did not involve wild animals.
Reporting on sex	No considers on sex in this study.
Field-collected samples	The study did not involve samples collected from the field.
Ethics oversight	This research complies with all relevant ethical regulations. All animal protocols were approved by Institutional Animal Care and Use Committees (IACUC) of Peking University, which are accredited by the Association for Assessment and Accreditation of Laboratory Animal Care International (AAALAC). All zebrafish experiments were approved and carried out in accordance with the Animal Care Committee at the Institute of Zoology, Chinese Academy of Sciences.

Note that full information on the approval of the study protocol must also be provided in the manuscript.

Flow Cytometry

Plots

Confirm that:

☒ The axis labels state the marker and fluorochrome used (e.g. CD4-FITC).

☒ The axis scales are clearly visible. Include numbers along axes only for bottom left plot of group (a 'group' is an analysis of identical markers).

☒ All plots are contour plots with outliers or pseudocolor plots.

☒ A numerical value for number of cells or percentage (with statistics) is provided.

Methodology

Sample preparation	Cells were digested and resuspended in culture medium, filtered through 70 um nylon mesh and then analyzed or sorted.
Instrument	FACSAria Ⅲ and LSRFortessa (BD Biosciences)
Software	FlowJo V10
Cell population abundance	Flow cytometry was performed on bulk cells. 10000 cells analyzed for each condition.
Gating strategy	The gating and sorting strategy is illustrated in the figures. Cells were gated using FSC/SSC to exclude debris and boublets. GFP-positivity is defined by comparing with no GFP-expressed cells.

☒ Tick this box to confirm that a figure exemplifying the gating strategy is provided in the Supplementary Information.
