# Supplementary figures and images for: Computational prediction and experimental validation identify functionally conserved lncRNAs from zebrafish to human
Source: Nat Genet. 2024 Jan 9;56(1):124–35. doi: 10.1038/s41588-023-01620-7 (PMC10786727; doi:10.1038/s41588-023-01620-7)

Unprocessed gels for Fig. 5b

RP1-212P9.3

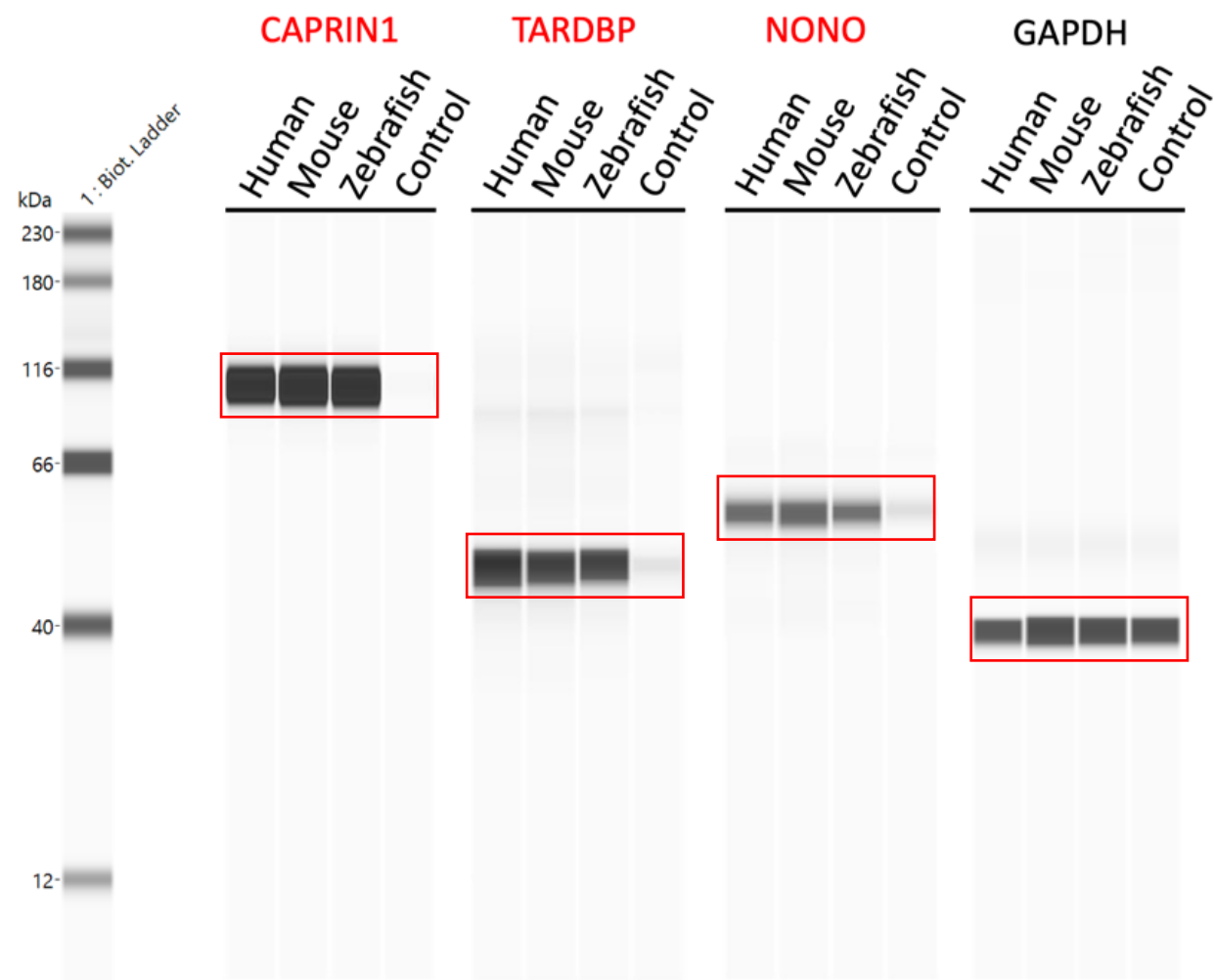

Supplement: Supplementary file 6 — Unprocessed scans of gels for Fig. 5b. [file 41588_2023_1620_MOESM6_ESM.pdf]

Unprocessed gels for Extended Data Fig. 9d

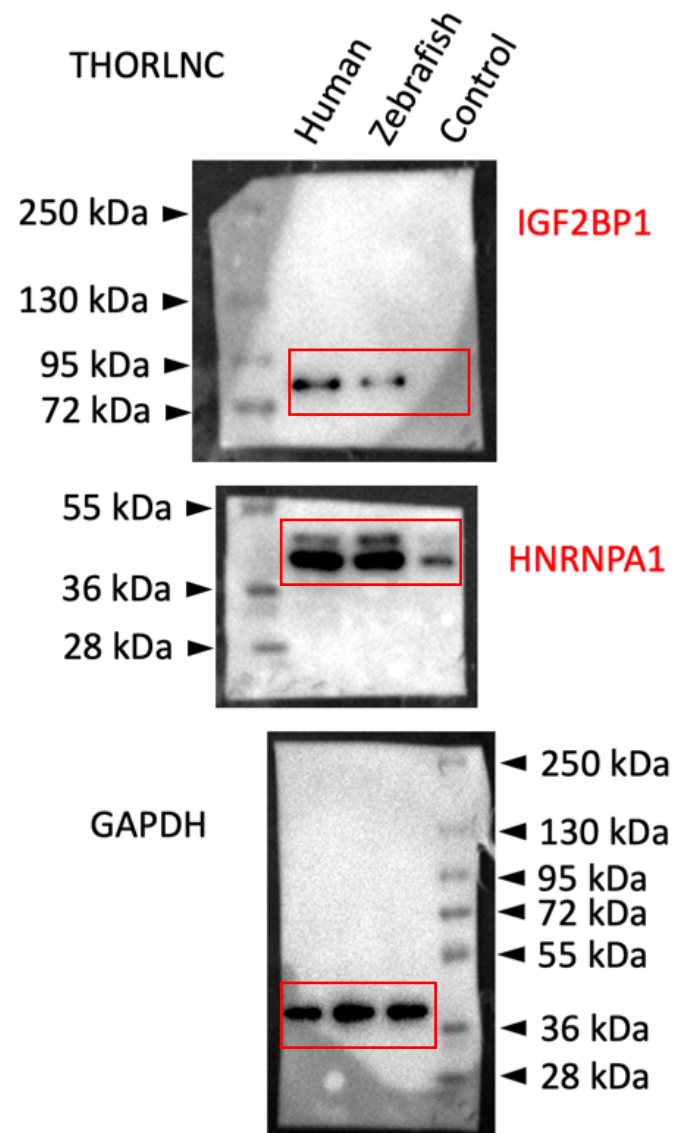

Supplement: Supplementary file 9 — Unprocessed scans of gels for Extended Data Fig. 9d. [file 41588_2023_1620_MOESM9_ESM.pdf]
