# Supplementary material for: Computational prediction and experimental validation identify functionally conserved lncRNAs from zebrafish to human
Source: Nat Genet. 2024 Jan 9;56(1):124–35. doi: 10.1038/s41588-023-01620-7 (PMC10786727; doi:10.1038/s41588-023-01620-7)
Supplement: Supplementary file 7 — Unprocessed scans of gels for Extended Data Fig. 5c. [file 41588_2023_1620_MOESM7_ESM.pdf]

Unprocessed gels for Extended Data Fig. 5c

MW: Trans 5K

1,500bp

500bp

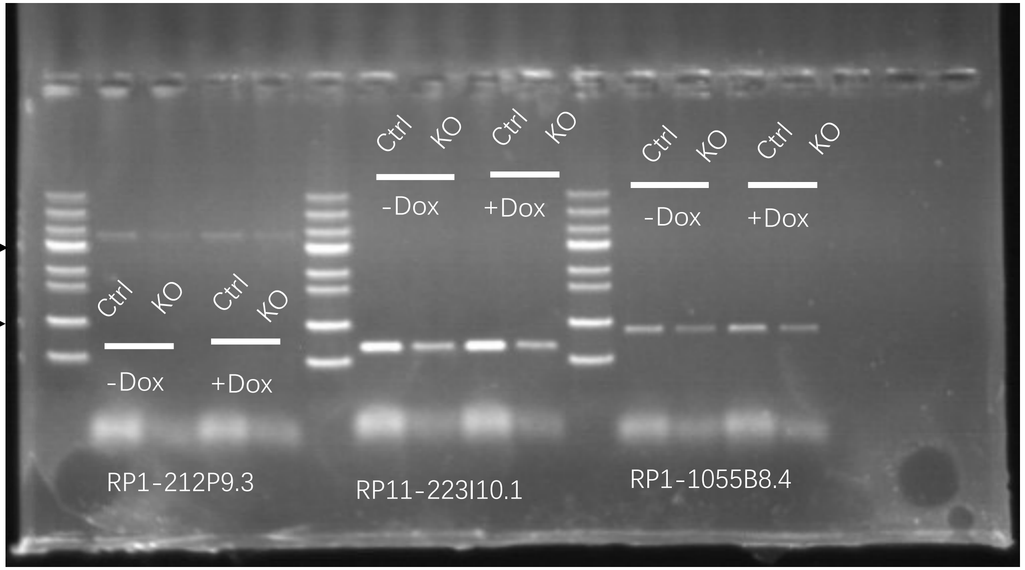

MW: Trans 5K

1,500bp

500bp

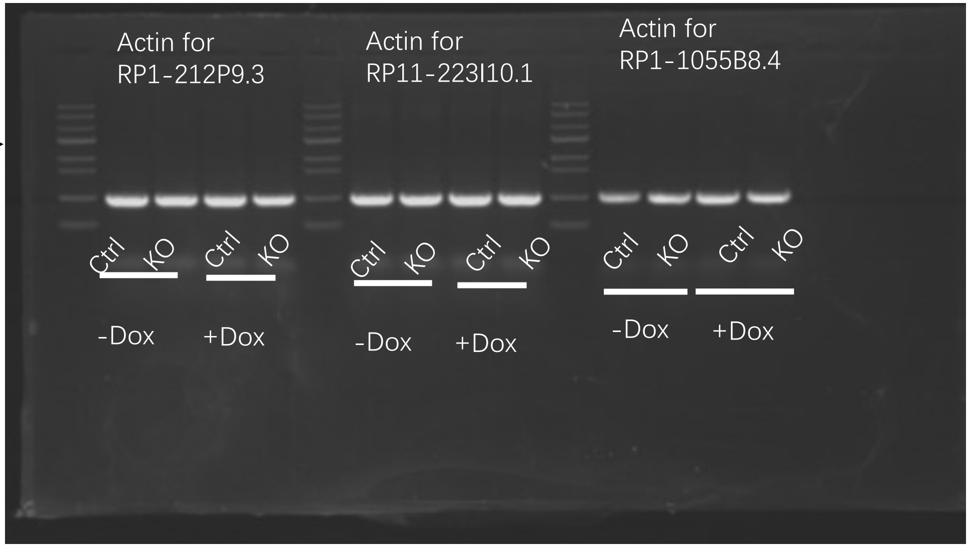

MW: Trans 5K

1,500bp

500bp

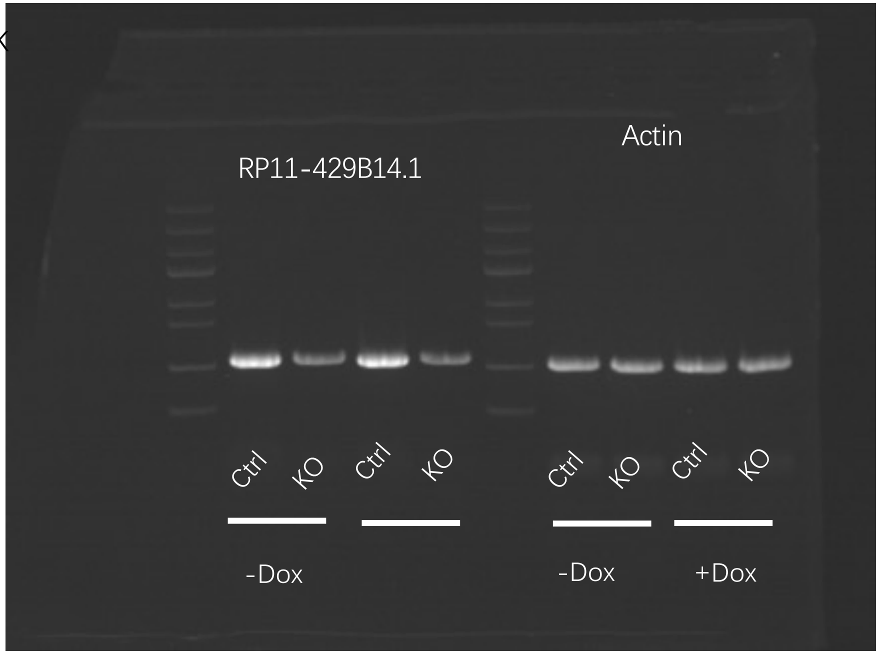

Replicate 1 (shown in this paper)

MW: Trans 2K plus

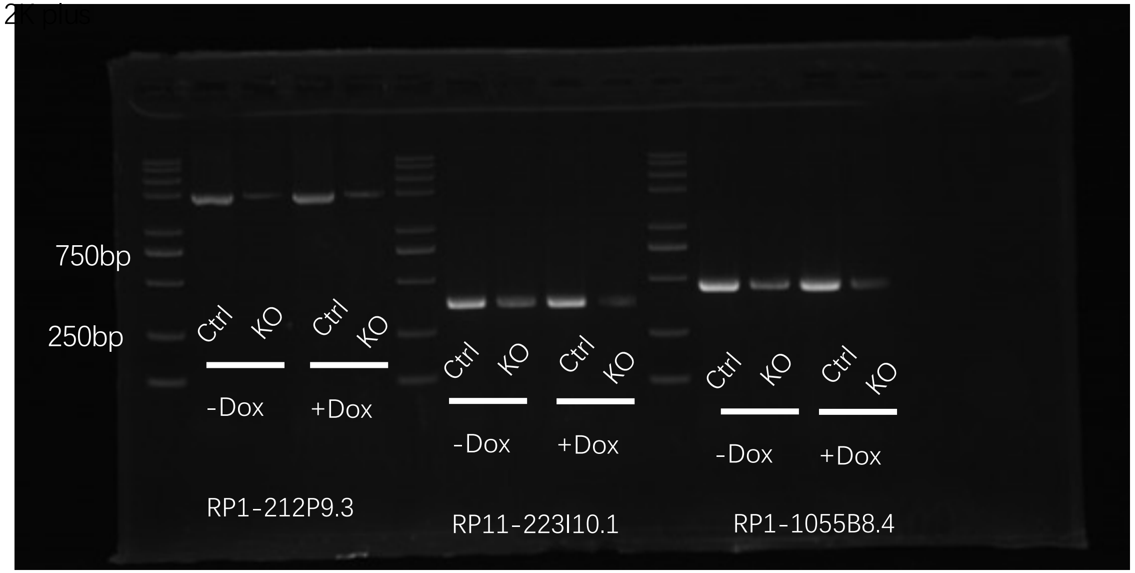

MW: Trans 2K plus

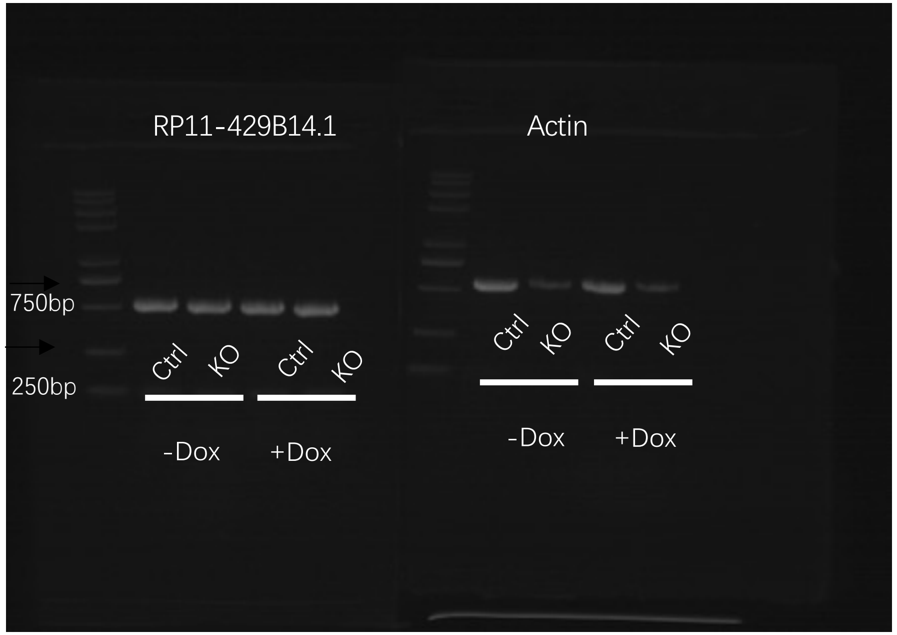

MW: Trans 2K plus

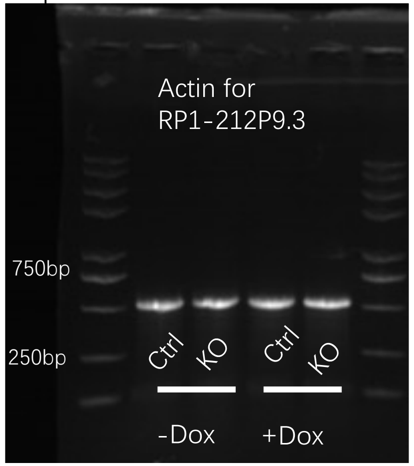

MW: Trans 2K plus

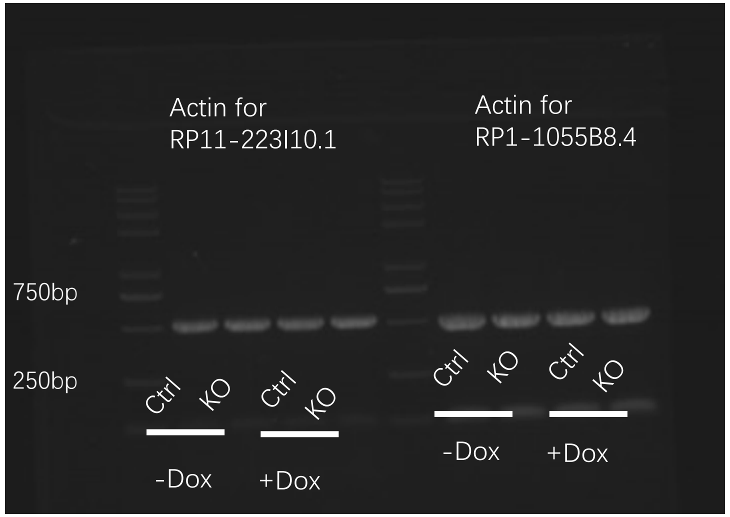

Replicate 2

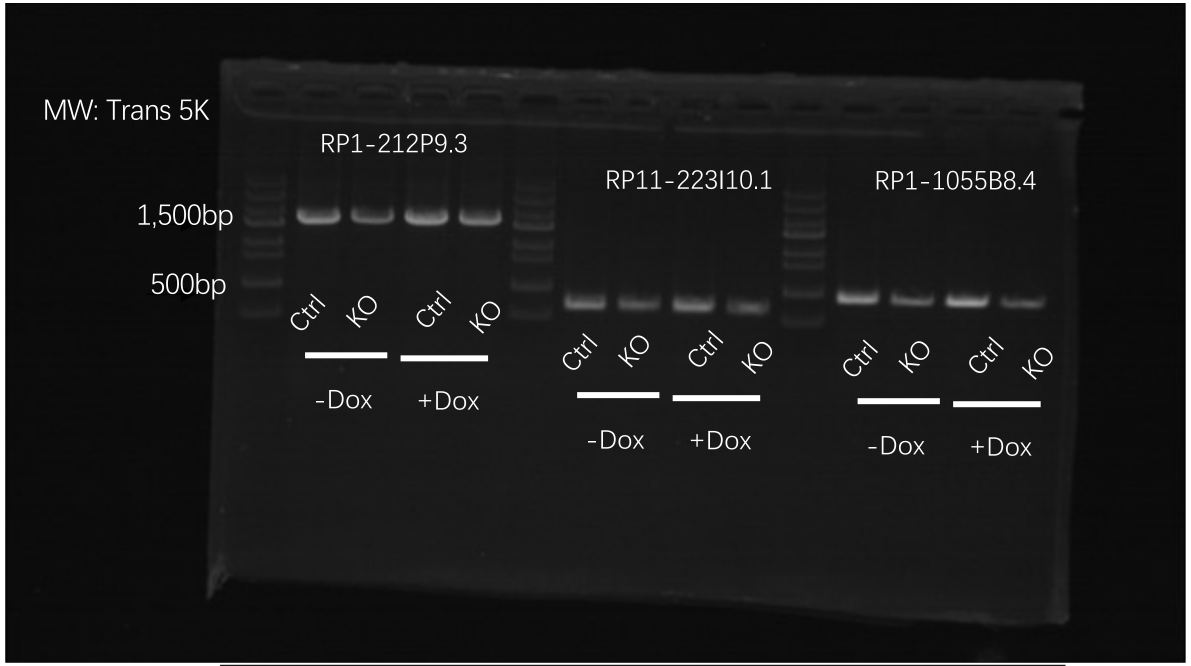
MW: Trans 5K
RP1-212P9.3
RP11-223I10.1
RP1-1055B8.4
1,500bp
500bp
Ctrl KO Ctrl KO
-Dox +Dox
Ctrl KO Ctrl KO
-Dox +Dox
Ctrl KO Ctrl KO
-Dox +Dox

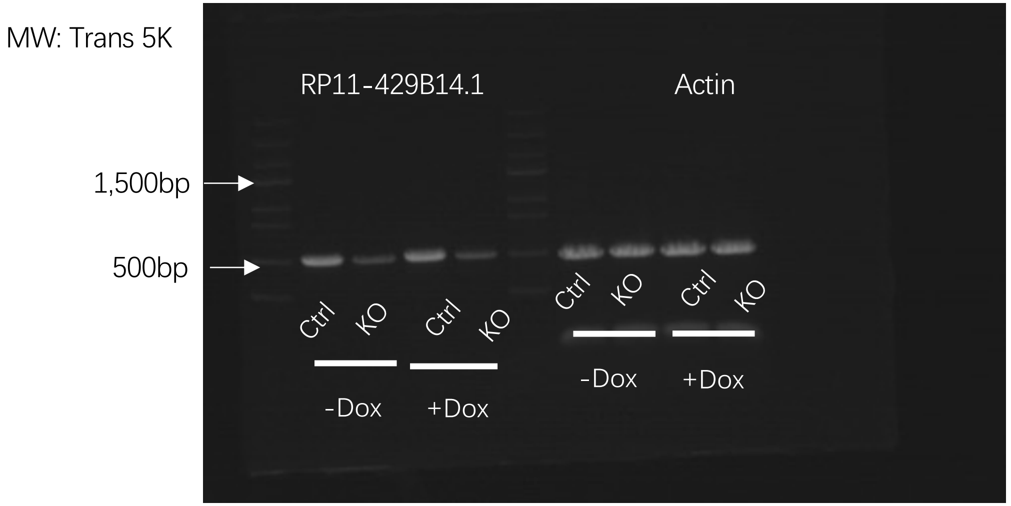
MW: Trans 5K
RP11-429B14.1
Actin
1,500bp
500bp
Ctrl KO Ctrl KO
-Dox +Dox
Ctrl KO Ctrl KO
-Dox +Dox

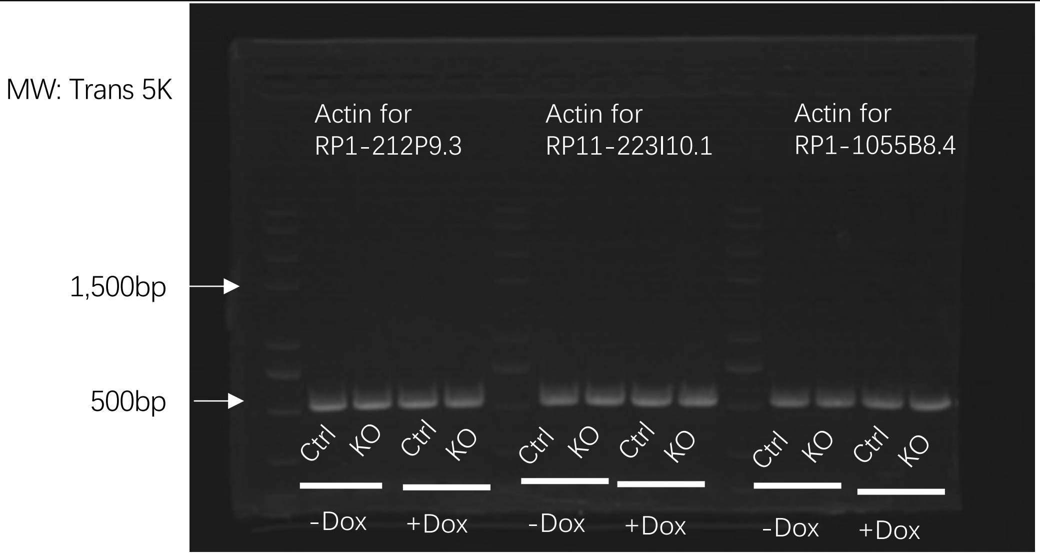
MW: Trans 5K
Actin for RP1-212P9.3
Actin for RP11-223I10.1
Actin for RP1-1055B8.4
1,500bp
500bp
Ctrl KO Ctrl KO
-Dox +Dox
Ctrl KO Ctrl KO
-Dox +Dox
Ctrl KO Ctrl KO
-Dox +Dox

Replicate 3
