# Supplementary material for: Computational prediction and experimental validation identify functionally conserved lncRNAs from zebrafish to human
Source: Nat Genet. 2024 Jan 9;56(1):124–35. doi: 10.1038/s41588-023-01620-7 (PMC10786727; doi:10.1038/s41588-023-01620-7)
Supplement: Supplementary file 8 — Unprocessed scans of gels for Extended Data Fig. 7d. [file 41588_2023_1620_MOESM8_ESM.pdf]

Unprocessed gels for Extended Data Fig. 7d

MW: Trans 2K plus

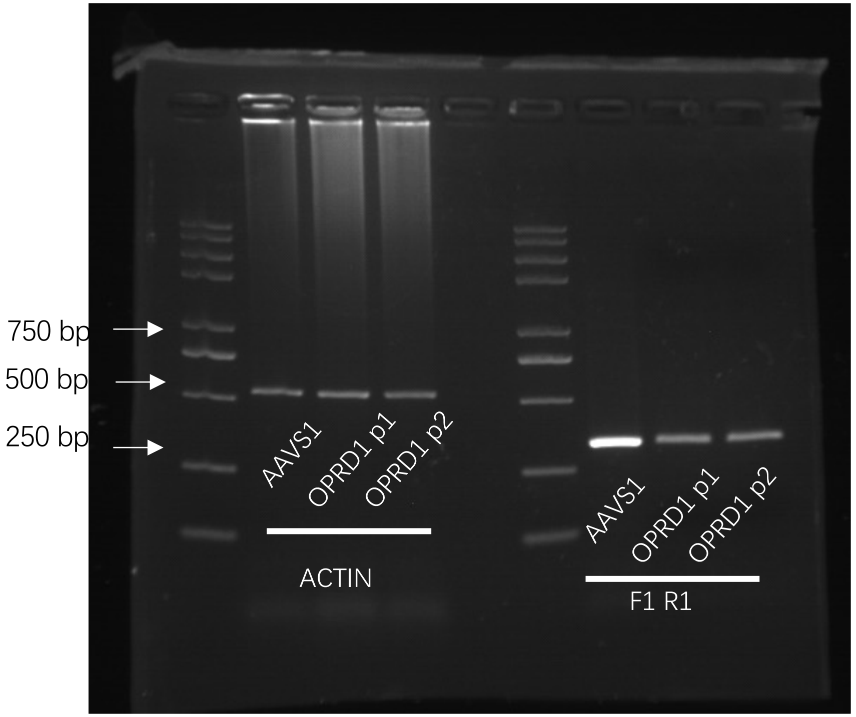

Replicate 1 (shown in this paper)

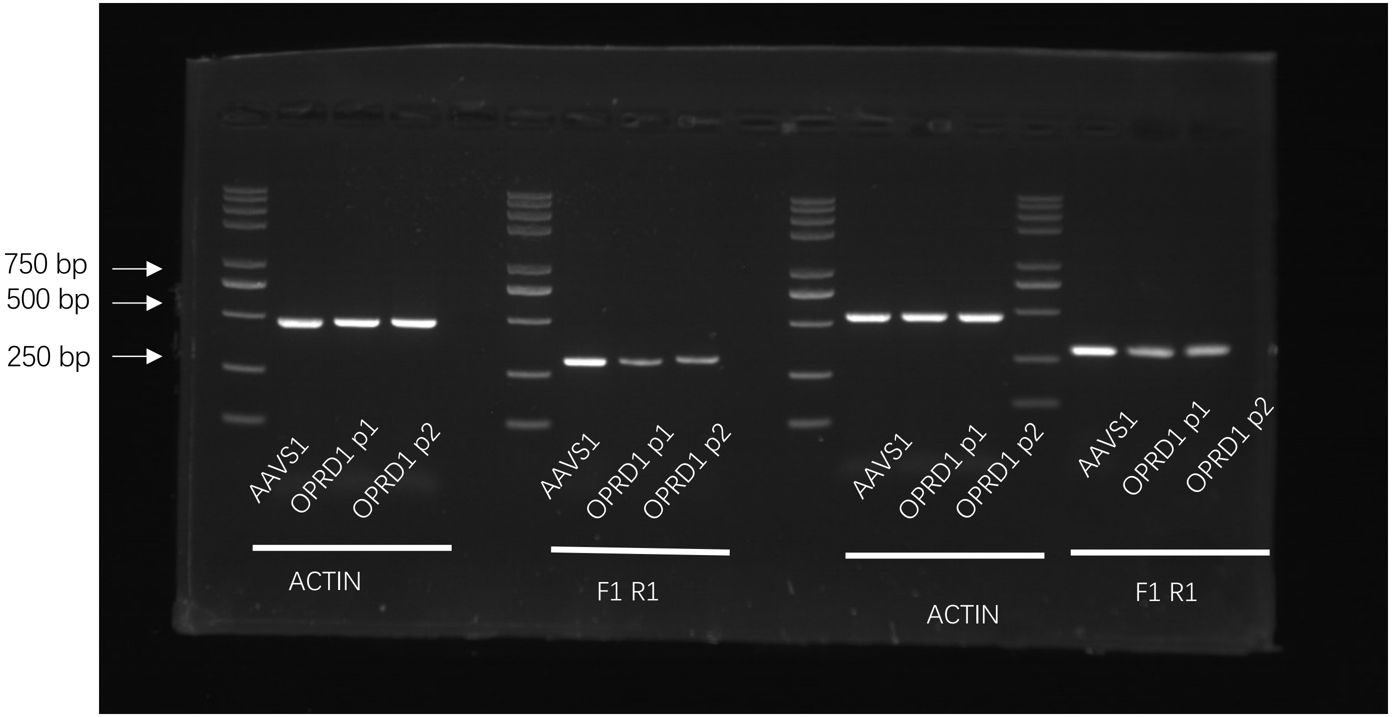

Replicate 2 and 3
